# A novel role of Krüppel-like factor 8 as an apoptosis repressor in hepatocellular carcinoma

**DOI:** 10.1186/s12935-020-01513-3

**Published:** 2020-08-28

**Authors:** Ming-Da Wang, Hao Xing, Chao Li, Lei Liang, Han Wu, Xin-Fei Xu, Li-Yang Sun, Meng-Chao Wu, Feng Shen, Tian Yang

**Affiliations:** 1 Department of Hepatobiliary Surgery, Eastern Hepatobiliary Surgery Hospital, Second Military Medical University (Navy Medical University), No. 225, Changhai Road, Shanghai, 200438 China; 2 Department of Clinical Medicine, Second Military Medical University (Navy Medical University), Shanghai, China

**Keywords:** Hepatocellular carcinoma, KLF8, Apoptosis, H3K27 acetylation, HMGA2, MMP7

## Abstract

**Background:**

Krüppel-like factor 8 (KLF8), a cancer-promoting factor that regulates critical gene transcription and cellular cancer-related events, has been implicated in tumor development and progression. However, the functional role of KLF8 in the pathogenesis of hepatocellular carcinoma (HCC) remains largely unknown.

**Methods:**

The gene expression patterns and genome-wide regulatory profiles of HCC cells after KLF8 knockout were analyzed by using RNA sequencing (RNA-seq) and chromatin immunoprecipitation sequencing (ChIP-seq) of histone H3 lysine 27 acetylation (H3K27ac) combined with bioinformatics analysis. Transcription factor-binding motifs that recognized by KLF8 were evaluated by motif analysis. For the predicted target genes, transcriptional changes were examined by ChIP, and loss of function experiments were conducted by siRNA transfection.

**Results:**

KLF8 functioned as a transcription repressor in HCC and mainly regulated apoptotic-related genes directly. A total of 1,816 differentially expressed genes after KLF8 knockout were identified and significantly corresponded to global changes in H3K27ac status. Furthermore, two predicted target genes, high-mobility group AT-hook 2 (HMGA2) and matrix metalloproteinase 7 (MMP7), were identified as important participants in KLF8-mediated anti-apoptotic effect in HCC. Knockout of KLF8 enhanced cell apoptosis process and caused increase in the associated H3K27ac, whereas suppression HMGA2 or MMP7 attenuated these biological effects.

**Conclusions:**

Our work suggests a novel role and mechanism for KLF8 in the regulation of cell apoptosis in HCC and facilitates the discovery of potential therapeutic targets for HCC treatment.

## Introduction

Hepatocellular carcinoma (HCC) is the sixth most prevalent malignancy and represents the third highest cause of cancer-related death worldwide, with an age-adjusted incidence of 10.1 new cases per 100,000 individuals annually [1]. Although significant improvements regarding the diagnosis and treatment approaches have been made in recent years, HCC is still difficult to cure due to late diagnosis, drug resistance, and high rate of postsurgical recurrence [2, 3]. Despite great efforts in exploring the pathogenesis of HCC during last decades, the detailed mechanisms of HCC have not been fully clarified. Therefore, better understanding of the molecular mechanisms underlying HCC progression is essential for developing more effective therapeutic approaches and finding novel molecular targets for HCC treatment [4, 5].

Krüppel-like factor 8 (KLF8) belongs to the KLF family of transcription factors, which shares the similar well-conserved zinc finger DNA-binding domains, and plays a critical role in controlling various cellular processes, such as cell cycle, proliferation, differentiation, and cell transformation [6–8]. KLF8 has been reported overexpressed in multiple human malignancies and emerged as a crucial regulator of tumorigenesis and progression [9–11]. Concrete evidence has indicated the oncogenic role of KLF8 in the pathogenesis of HCC [11]. Previous studies demonstrated that KLF8 was positively correlated with the metastatic potential of HCC and promoted HCC proliferation and invasion both in vitro and in vivo [10]. In addition, the prognostic significance of KLF8 expression in patients with HCC has also been revealed. Our previous work also confirmed the positive correlation between upregulation of KLF8 and poor prognosis of HCC patients, and identified the KLF8-mediated activation of Wnt/β-catenin signaling pathway as a novel regulatory mechanism underlying HCC tumorigenesis [12]. Recently, it has been documented that various signaling pathways were responsible for KLF8-drived HCC tumorigenesis and metastasis [13, 14]. However, the detailed regulatory mechanisms of KLF8, as well as its potential roles in the promotion of HCC progression remain largely unknown.

In the present study, we firstly investigated the functional role of KLF8 in HCC tumorigenesis and explored the gene expression profile, as well as involved signaling pathways by using RNA sequencing (RNA-seq) combined with bioinformatics analyses[15, 16]. Subsequently, we screened the relevant genes exhibiting significant changes by chromatin immunoprecipitation (ChIP) assay and identified high-mobility group AT-hook 2 (HMGA2) and matrix metalloproteinase 7 (MMP7) as important participants contributing to KLF8-mediated anti-apoptotic effect in HCC. Taken together, our results provided a novel mechanistic insight into the critical role of KLF8 in suppressing cell apoptosis during HCC progression, suggesting a potential therapeutic target for HCC treatment.

## Materials and methods

### Cell cultures

Human HCC cell lines LM3 and Huh-7 were purchased from Cell Bank of Typical Culture Preservation Commission (Chinese academy of sciences, Shanghai, China), and were routinely cultured at 37 °C in an atmosphere containing 5% CO2 in Dulbecco’s modified Eagle medium (DMEM, Gibco, Life Technologies) supplemented with 10% Fetal Bovine Serum (FBS, Gibco) and 1% Penicillin–Streptomycin. KLF8 was knocked out in HCC cell line LM3 (KLF8^KO^-LM3) by using the CRISPR (clustered regularly interspaced short palindromic repeats)/Cas9 technique according to manufacturer's protocols [17]. The human KLF8 sequence was cloned and ligated into the GV303 plasmid (GeneChem, Shanghai). The titers of concentrated viral particles ranged between 5 × 10^8^ and 1 × 10^9^ transducing units/ml. Lentiviral particles containing human KLF8 gene were constructed and added to Huh-7 cells at a multiplicity of infection of 20 in the presence of polybrene (5 μg/ml) for 8 h, and the supernatant was replaced with normal cultured medium 12 h after infection. Small interfering (si)RNAs for HMGA2 and MMP7, as well as negative control siRNAs were purchased from Shanghai Biotend Company. For siRNA transfection, 100 nM siRNAs were added into HCC cells maintaining in 6-well plate with INTERFERin transfection reagent (Polyplus) according to manufacturer’s instructions.

### RNA-seq

Total RNA was extracted from LM3 or KLF8^KO^-LM3 cells using TRIzol reagent (Invitrogen) according to the manufacturer's instructions. RNA quality was evaluated with a BioAnalyzer 2100 system (Agilent Technologies, USA). Small RNAs had linkers ligated to them and bar-coded cDNAs were prepared using a TruSeq Sample Prep Kit (Illumina, USA) following the manufacturer’s instructions. Individual libraries were analyzed for the presence of linked cDNA at the appropriate size (140–150 bp) as determined by the BioAnalyzer. Subsequently, the amplified cDNA constructs were purified from agarose gel in preparation for sequencing analysis using the Illumina HiSeq 2500 platform (Illumina, CA, USA) according to the manufacturer’s instructions at the Shanghai Biotechnology Corporation.

The RNA sequence reads were pre-processed using the FASTXTool kit to exclude low-quality reads (ambiguous N, quality < 10 nt, and length < 18 nt) and 3′ adapter, 5′ adapter and poly A sequences). Further annotation analyses were performed using the commercial software CLC Genomic Workbench 5.5. After all annotation steps, the sequencing libraries were used for size distribution and saturation analysis.

### Bioinformatics analysis

We employed MACS2 (https://pypi.python.org/pypi/MACS2) to identify histone H3 lysine 27 acetylation (H3K27ac) peaks in both LM3 and KLF8^KO^-LM3 cells. We set H3K27ac in KLF8^KO^-LM3 cells as treatment and H3K27ac in LM3 cells as control to detect KLF8^KO^-LM3 specific peaks (*P*-value was set as 0.00001). BETA was used to integrate differential expression calculated by Deseq2 (https://www.bioconductor.org) with KLF8^KO^-LM3 specific peaks (-d 2000 –df 0.05) to obtain genes whose transcription levels are regulated by KLF8. We run FIMO (https://meme.sdsc.edu) over all of promoters for hits of KLF8 motif to further identify putative direct targets of KLF8 (Additional file 1: Table S1).

### ChIP assay

ChIP assay was performed using an EZ ChIP™ Chromatin Immunoprecipitation Kit (17–371, Millipore) following the manufacturer’s protocol. Quantitative real-time PCR was conducted to detect DNA fragments binding with KLF8. Primers were designed to detect predicted promoters or enhancers (Additional file 2: Table S2). The ratio of DNA binding with KLF8 versus total DNA was calculated.

### Western blotting

Primary cell cultures were homogenized in NP40 buffer (Beyotime, China) supplemented with PMSF (1 mM, Sigma-Aldrich). The cell lysates were subjected to western blotting using the anti-Caspase3 (1:1000, 9662 s, Cell Signaling Technology), anti-KLF8 (1:500, 2,101,276, Sigma-Aldrich), anti-Flag (1:1000, 14,793, Cell Signaling Technology) and HRP-conjugated anti-GAPDH (Kangcheng, Shanghai, China) antibodies. The protein bands were analyzed using Image Lab analysis (Bio-Rad).

### Apoptosis assay

For apoptosis analysis, LM3 or KLF8^KO^-LM3 cells were stained with FITC-Annexin-V and PI using a Cell Apoptosis Analysis Kit (Sungene, Tianjin) according to the manufacturer’s instructions. Annexin-V^+^/PI^−^ cells were considered as cells in apoptosis.

### Cell proliferation assay

Cell proliferation assay was carried out using CCK8 cell proliferation assays kit (Dojindo, Japan) according to the manufacturer’s instruction. Briefly, Huh-7 cells transfected with KLF8 or negative control vectors or LM3/KLF8^KO^-LM3 cells were placed into 96-well plate. After 18 h after adding 20 μM camptothecin (sigma-aldrich), 10 μl CCK-8 assay solution was added. Then, after incubation for another 1 h, optical density (OD) at 450 nm was measured with an enzyme immunoassay analyzer (Thermo Fisher Scientific, Inc., Waltham, MA, USA) to estimate cell proliferation.

### Statistical analysis

Gene ontology (GO) enrichment analyses were performed with the DAVID (Database for Annotation, Visualization, and Integrated Discovery) database (https://david.abcc.ncifcrf.gov). Kyoto Encyclopedia of Genes and Genomes (KEGG) pathway analyses were performed to determine the significant pathways (https://www.kegg.jp/kegg/pathway.html). The cluster Profiler Package V3.6.0 was used to identify and visualize the GO terms and KEGG pathway enrichments. A two-tailed Student’s t-test was applied for statistical comparison of 2 groups. The data are presented as the mean ± standard deviation (SD), unless otherwise indicated and “mean ± SD” generally represents biological replicates. All statistical analyses were performed using the SPSS software version 25.0 (SPSS, Chicago, IL, USA) and R Project for Statistical Computing (version 3.6.3, the University of Auckland, New Zealand). A *P* < 0.05 was considered statistically significant.

## Results

### Knockout of KLF8 promoted cell apoptosis via up-regulating the expression of apoptotic-related genes

**Fig. 1:**
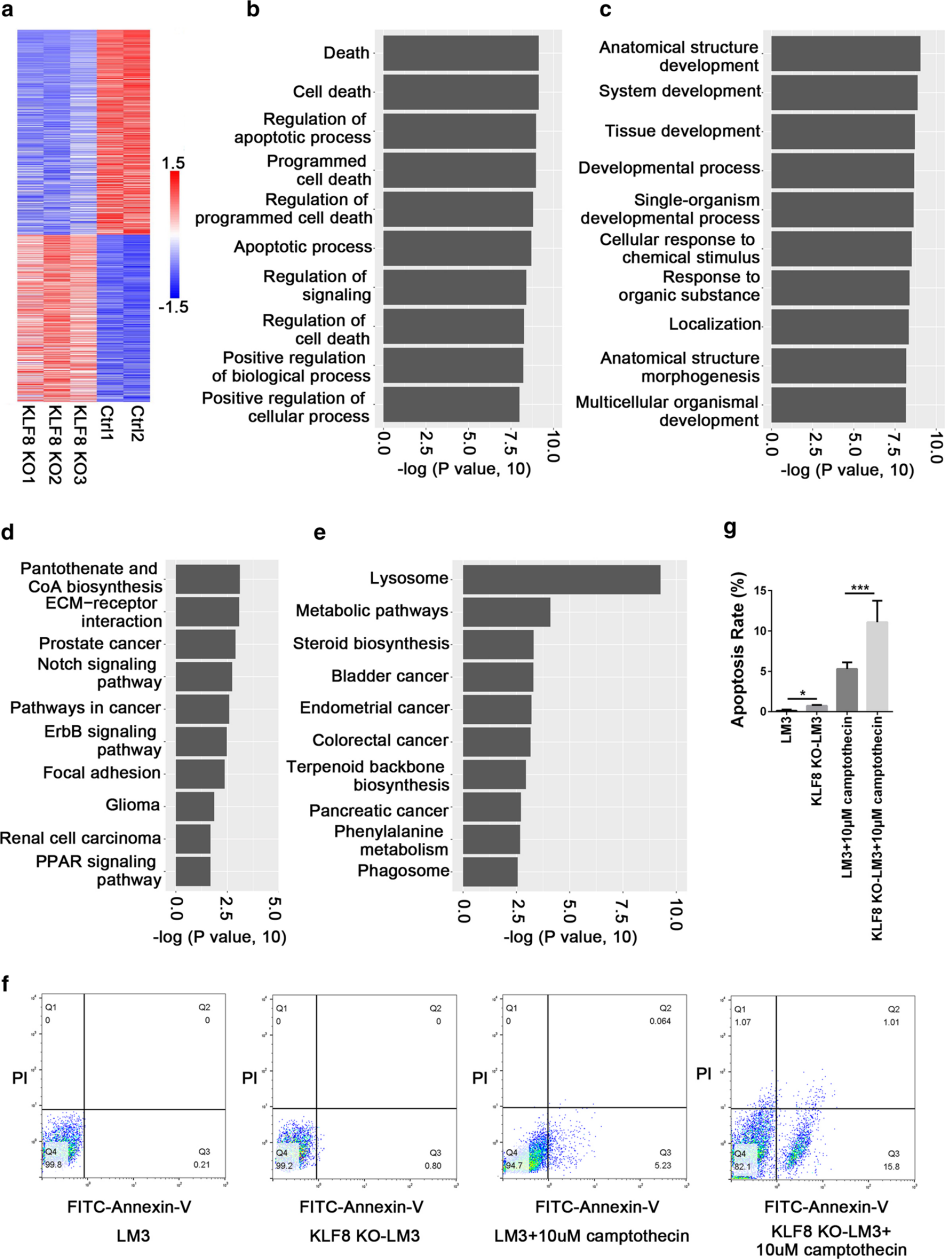
Knockout of KLF8 promoted cell apoptosis via up-regulating the expression of apoptotic-related genes. **a** Hierarchical clustering analysis of genes that were significantly up- or down-regulated genes between KLF8^KO^-LM3 cells and control. A cut off of log2 fold Change ≤ -1.5 and ≥ 1.5 was set (*P* < 0.05). Expression values were represented in colors of red and blue, which indicated high and low relative expression value across all samples (log scale 2, from 1.5 to − 1.5), respectively. **b**, **c** GO analysis associating genes up-regulated (**b**) or down-regulated (**c**) with GO categories. Significant GO terms influenced by knockout of KLF8 in LM3 cells. The vertical axis represents the GO category, and the horizontal axis is the log of the *P* value. **d**, **e** KEGG analysis associating genes up-regulated (**d**) or down-regulated (**e**) in LM3 control or KLF8^KO^-LM3 cells. The vertical axis is the pathway category, and the horizontal axis represents the log of the *P* value. **f** Representative flow cytometry analysis after Annexin-V/PI staining in LM3 control or KLF8^KO^-LM3 cells after exposure to 10 μM camptothecin. **g** Apoptosis rate of LM3 control or KLF8^KO^-LM3 cells in (**f**). Experiments were performed in triplicate and all data are shown as Mean ± SD. * *P* < 0.05 and *** *P* < 0.001

To obtain the insight into the role of KLF8 on gene expression in HCC cells, we firstly established a stable KLF8 knockout HCC cell line LM3 (KLF8^KO^-LM3) using CRISPR/Cas9 technology. The knockout effect was confirmed by Western blotting (Additional file 3: Fig. S1A). Next, we used RNA-seq to screen differentially expressed genes in KLF8^KO^-LM3 cells compared to control cells. By using a 1.5-log2 fold cut-off with a *P* value < 0.05, a total of 1,816 genes were identified to be significantly differentially expressed between the two cell lines (Fig. 1a), indicating that KLF8 possessed a general and critical influence on the gene expression profile in HCC cells. To characterize the function roles of these differentially expressed genes, we performed GO and KEGG pathway analyses and found that most of the top GO terms for biological processes enriched by up-regulated genes after KLF8 knockout were closely relative to cell apoptosis (Fig. 1b), while the down-regulated ones were largely associated with various development processes of tissues or organs (Fig. 1c). In KEGG analysis, signaling pathways showing significant enrichment after KLF8 knockout mainly corresponding to the biosynthesis of metabolites, lysosome, ECM-receptor interaction, and Notch pathway (Fig. 1d, e).

Given that knockout of KLF8 mainly promoted the expression of apoptotic-related genes in HCC, we further examined whether down-regulation of KLF8 promoted cell apoptosis. As expected, the flow cytometry results revealed that knockout of KLF8 significantly promoted cell apoptosis after exposure to camptothecin (LM3 v.s. KLF8 KO-LM3, *P* = 0.011; LM3 + cam v.s. KLF8 KO-LM3 + cam, *P* < 0.001) (Fig. 1f, g). The CCK8 experiments also showed a consistent result with enhanced ability of cell proliferation after KLF8 knockout (Additional file 3: Fig. S2). Taken together, these results suggested that KLF8 was involved in cell apoptosis and probably functioned as a repressor of apoptotic-related genes in HCC.

### Knockout of KLF8 changed the acetylation of H3K27 in HCC cells

**Fig. 2:**
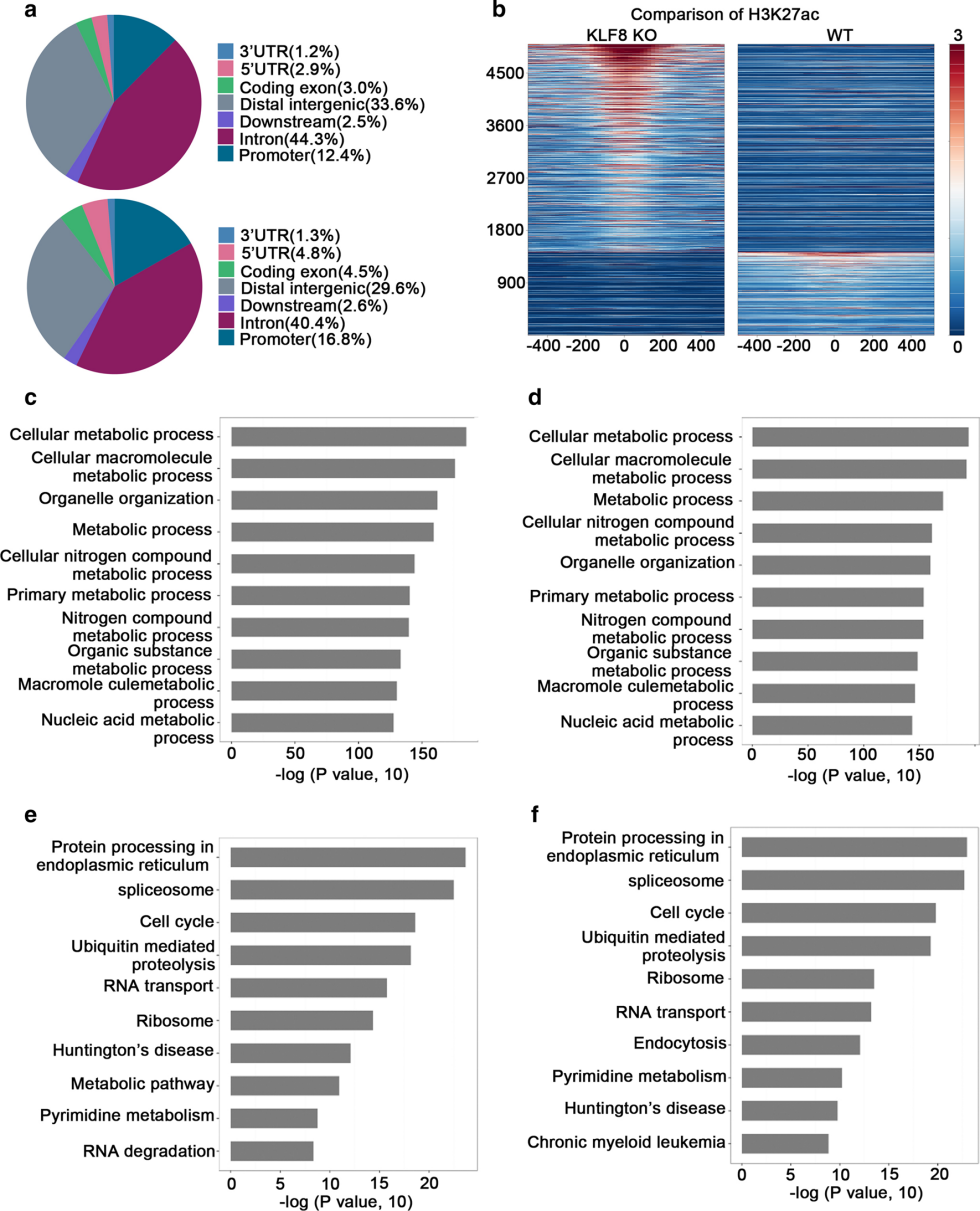
Knockout of KLF8 changed the H3K27 acetylation genes in HCC cells. **a** Distribution of DNA fragments obtained by anti-H3K27ac ChIP-seq in KLF8^KO^-LM3 (up) or LM3 control (down) cells. **b** Comparison of H3K27ac between KLF8^KO^-LM3 (left) and LM3 (right) cells. H3K27ac ChIP-seq signals are plotted on center-aligned peaks from either KLF8^KO^-LM3 or LM3. K-Means cluster is employed to separate peaks with more enrichment in KLF8^KO^-LM3 and those with more enrichment in LM3. The significant enrichment of H3K27ac upon KLF8 knockout suggests repressive role of KLF8 in gene regulation. **c**, **d** GO analysis of H3K27ac genes in KLF8^KO^-LM3 (**c**) or LM3 (**d**) cells. The vertical axis represents the GO category, and the horizontal axis is the log of the *P* value. **e**, **f** KEGG analysis of H3K27ac genes in KLF8^KO^-LM3 (**e**) or LM3 (**f**) cells. The vertical axis is the pathway category, and the horizontal axis represents the log of the *P* value

As a transcription factor, we intended to determine whether KLF8 could directly regulate the expression of apoptotic-related genes. However, owing to the lack of qualified anti-KLF8 antibody, we failed to analyze transcriptome and genome-wide regulatory profiles of KLF8^KO^-LM3 cells by using ChIP coupled with high-throughput sequencing (ChIP-seq). It is well known that transcription regulation of gene expression is often accompanied with histone modification of related enhancers or promoters [18]. Among the epigenomic profiling, H3K27ac is a typical indicator of active DNA regulatory regions and plays a fundamental role in the regulation of gene transcription [19–21]. To identify those genes that were directly transcriptional regulated by KLF8, we performed the ChIP-seq assay using the anti-H3K27ac antibody, which bypasses the need for KLF8 antibody with ChIP efficiency. As shown in Fig. 2a, we mapped differential H3K27ac profiles in DNA regulatory regions in both LM3 and KLF8^KO^-LM3 cells. The comparison of H3K27ac between LM3 and KLF8^KO^-LM3 cells showed that knockout of KLF8 contributed to a much higher H3K27ac level, exhibiting the inhibitory role of KLF8 in regulation of gene transcription in HCC (Fig. 2b). However, because of the huge background transcriptional activity, GO analysis of H3K27ac genes showed no significant difference in the major functional subgroups between LM3 and KLF8^KO^-LM3 cells (Fig. 2c, d). KEGG pathway analysis also demonstrated no significant difference between the two groups (Fig. 2e, f). These results indicated that KLF8 functioned as a transcription repressor in HCC, but not significantly affected the major biological processes.

### Knockout of KLF8 increased the H3K27ac level of apoptotic-related genes

**Fig. 3:**
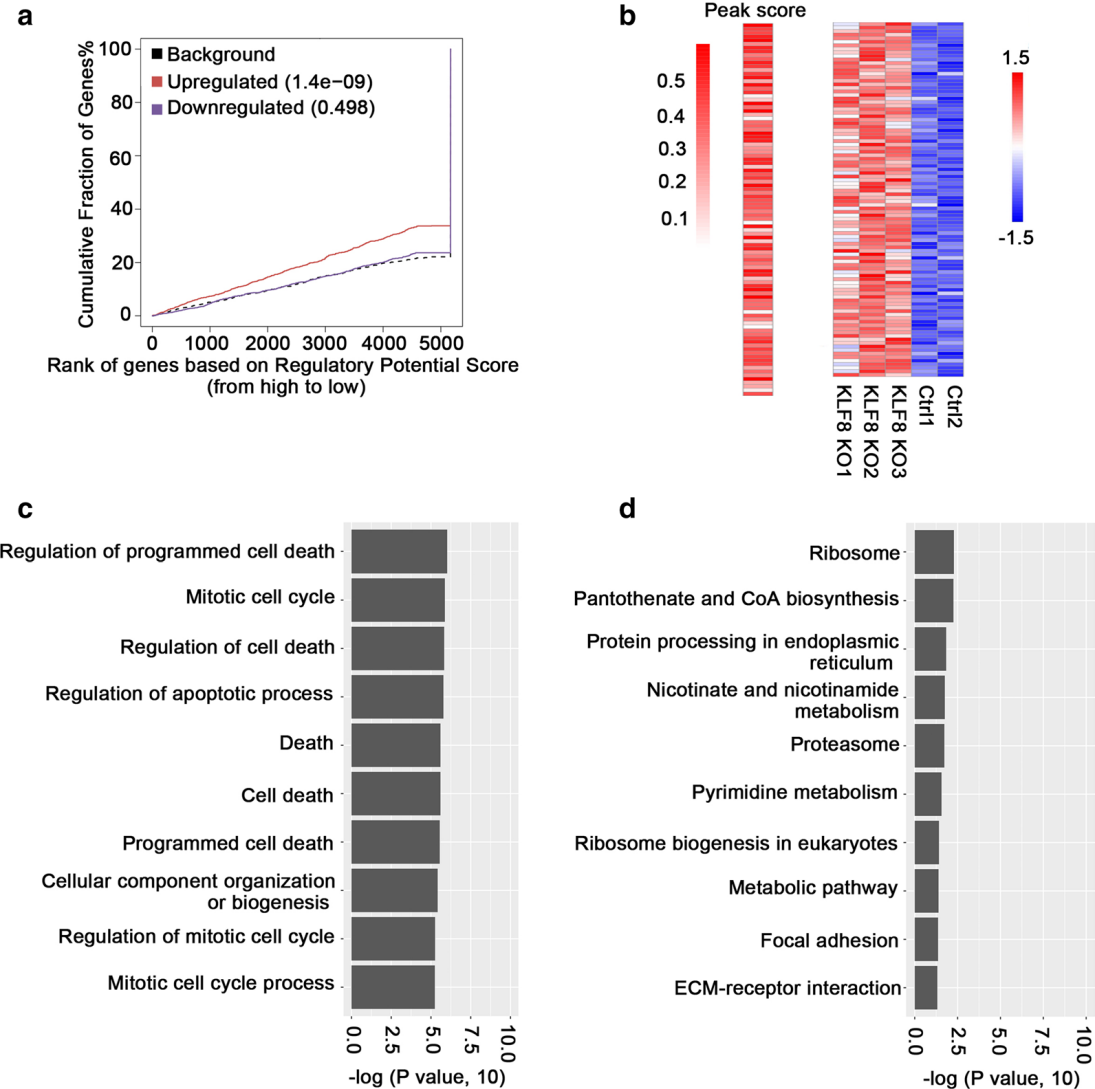
Knockout of KLF8 increased the H3K27ac level of apoptotic-related genes. **a** BETA activating/repressive function prediction of the transcriptome and H3K27ac data from KLF8^KO^-LM3 or LM3 cells. The red and the purple lines represent the up-regulated and down-regulated genes, respectively. The dashed line indicates the non-differentially expressed genes as background. Genes are cumulated by the rank on the basis of the regulatory potential score from high to low. *P* values that represent the significance of the UP or DOWN group distributions in the brackets are compared with the NON group by the Kolmogorov–Smirnov test. **b** Left, the H3K27 acetylation peak score of the top 100 genes differentially expressed as well as H3K27ac changed between KLF8^KO^-LM3 cells and control. Right, the hierarchical clustering of the top 100 genes in left. **c** GO analysis of the overlapped genes (differentially expressed as well as H3K27ac changed) with GO categories. The vertical axis represents the GO category, and the horizontal axis is the log of the *P* value. **d** KEGG analysis of these overlapped genes. The vertical axis is the pathway category, and the horizontal axis represents the log of the *P* value

To explore whether the alternations of transcription corresponds with differences in H3K27ac, we performed a combined analysis of the RNA-seq and the H3K27ac ChIP-Seq. Analyses by integration of transcriptome and ChIP-seq data with “BETA” [22] showed that up-regulated genes were more likely the targets of KLF8 (Fig. 3a). We next screened the acetylated genes with high expression levels in the KLF8^KO^-LM3 cell line (Additional file 1: Table S1). 532 genes were identified and the “peak scores” of H3K27ac were calculated (Fig. 3b). GO analysis combining H3K27ac profiles and gene expression levels revealed genes to be related to cell apoptotic process (Fig. 3c), while KEGG analysis showed no special pathways were involved in tumor growth or migration (Fig. 3d). This conjoint analysis confirmed the accuracy of RNA-seq and also indicated that the role of KLF8 in HCC may be implemented by regulating the transcription of certain apoptotic-related genes.

### KLF8 directly regulated apoptotic-related genes

**Fig. 4:**
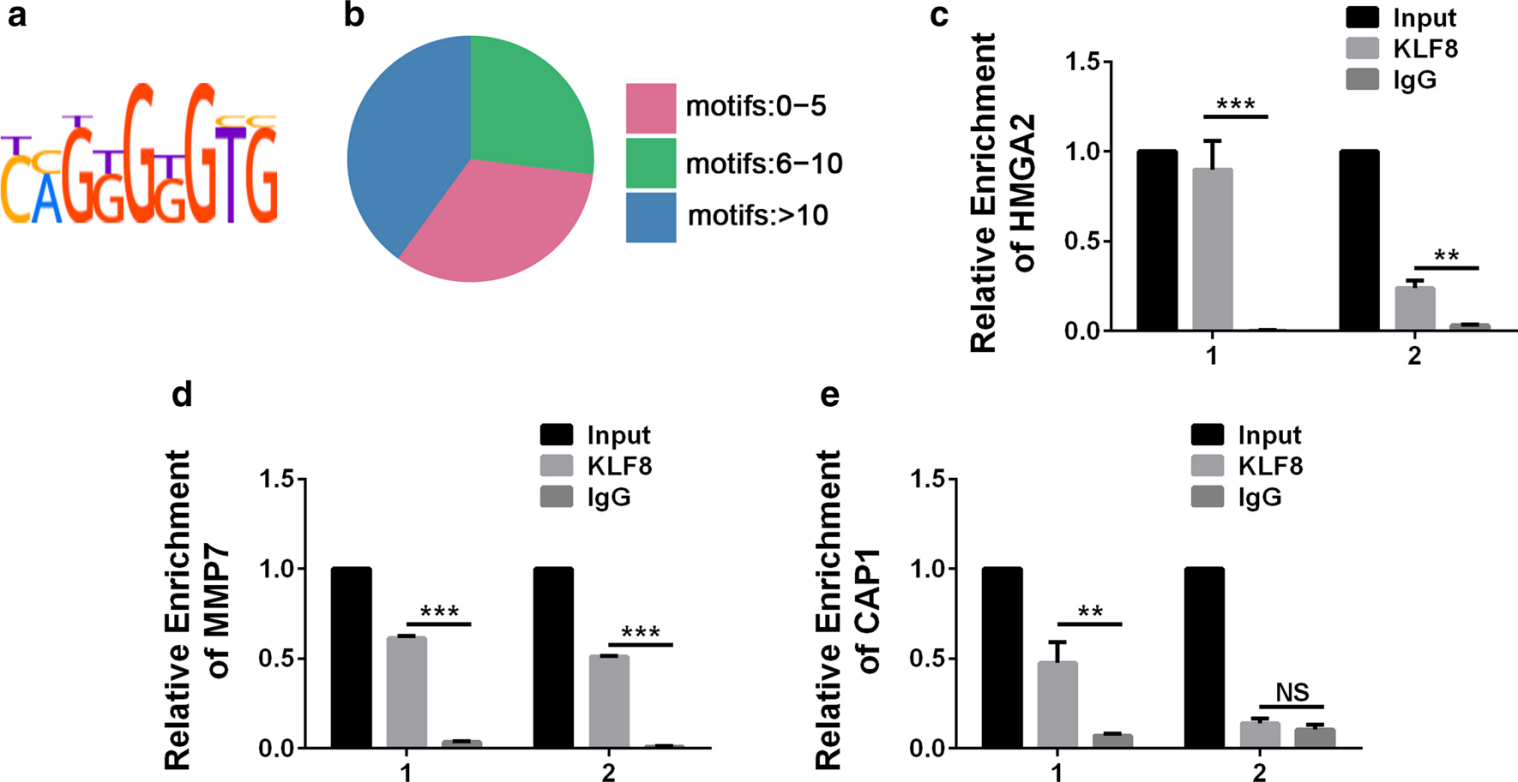
KLF8 directly regulated apoptotic-related genes. **a**. A schematic map of the motif recognized by KLF8. **b** Distribution of the number of KLF8 motifs in the overlapped genes (differentially expressed as well as H3K27ac changed). **c**–**e** ChIP analyses of overlapped apoptotic-related genes in LM3 (left) and Huh-7 (right) cells (**C**, HMGA2; **D**, MMP7; **E**, CAP1) with anti-KLF8 antibody. Experiments were performed in triplicate and all data are shown as Mean ± SD. ** *P* < 0.01 and *** *P* < 0.001

The combined analysis of the transcriptome and the anti-H3K27ac ChIP-seq filtered out 532 genes that may be regulated in the KLF8^KO^-LM3 cells. We then analyzed the promoters of these genes and identified the KLF8 recognition motif in 510 ones (Fig. 4a, b; Additional file 1: Table S1). The results demonstrated that anti-H3K27ac ChIP-seq was a reliable alternative method while KLF8 ChIP-seq data was unattainable. Next, we focused on the apoptotic-related genes which were probably the key participants in KLF8-mediated anti-apoptotic effects in HCC. Combining the results of motif analyses with the RNA-seq, we excluded the genes whose roles in HCC have been well-established and the ones with few motifs, and conducted ChIP experiments to verify the direct regulatory effect, as well as the specific binding between KLF8 and several apoptotic-related genes (KLF8 v.s. IgG in LM3 and Huh-7 cells: *P* < 0.001 and 0.003 for HMGA2; both *P* < 0.001 for MMP7; *P* = 0.004 and 0.056 for CAP1) (Fig. 4c–e; Additional file 2: Table S2). These results showed that KLF8 directly regulated the expression of apoptotic-related genes.

### HMGA2 and MMP7 participated in the anti-apoptotic effect mediated by KLF8

**Fig. 5:**
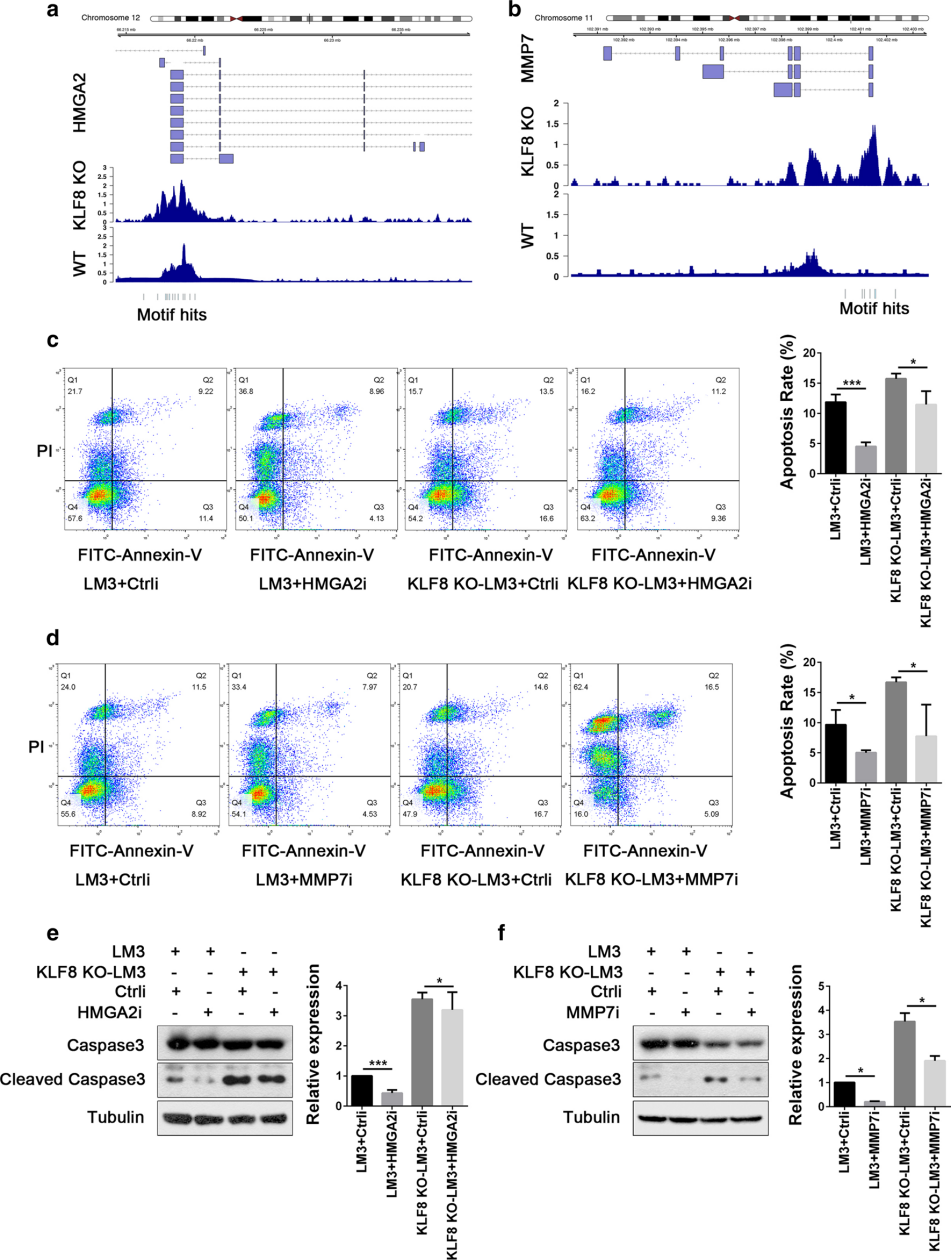
HMGA2 and MMP7 participated in the anti-apoptotic effect mediated by KLF8. **a**, **b** A schematic map of the location (up), H3K27ac (medium) and KLF8 motifs (down) of HMGA2 (**a**) and MMP7 (**b**) in the chromosome. **c** Flow cytometry analysis after Annexin-V/PI staining in LM3 (left) or KLF8^KO^-LM3 (right) cells transfected with HMGA2 siRNA (HMGA2i) or control siRNA (Ctrli). **d** Flow cytometry analysis after Annexin-V/PI staining in LM3 (left) or KLF8^KO^-LM3 (right) cells transfected with MMP7 siRNA (MMP7i) or Ctrli. For the right panels of C and D, experiments were performed in triplicate and all data are shown as Mean ± SD. * *P* < 0.05 and *** *P* < 0.001. **e** Western blot analysis of cleaved Caspase3 in LM3 or KLF8^KO^-LM3 cells transfected with HMGA2i or Ctrli. **f** Western blot analysis of cleaved Caspase3 in LM3 or KLF8^KO^-LM3 cells transfected with MMP7i or Ctrli. For E and F, representative results from 3 independent experiments are shown

Further, we intended to select certain molecules for functional verification from the apoptotic-related genes directly regulated by KLF8. HMGA2 and MMP7 exhibited concordant changes in gene expression and H3K27ac levels over promoter regions upon KLF8 knockout (Fig. 5a, b). Of these, HMGA2 was reported participating in the proliferation of many cancer types [23, 24], and MMP7 was found affecting the apoptotic process mediated by FAS/FASL system as well as N-cadherin [25, 26]. To further investigate their functional roles in cell apoptosis, the loss of function experiments were performed by transfecting specific siRNAs targeting HMGA2 or MMP7. As shown in Fig. 5c, d, knockout of KLF8 promoted cell apoptosis, whereas suppression of HMGA2 or MMP7 significantly attenuated this pro-apoptotic effect in KLF8^KO^-LM3 cells (LM3 + Ctrli v.s. LM3 + HMGA2i, *P* < 0.001; KLF8 KO-LM3 + Ctrli v.s. KLF8 KO-LM3 + HMGA2i, *P* = 0.015; and LM3 + Ctrli v.s. LM3 + MMP7i, *P* = 0.035; KLF8 KO-LM3 + Ctrli v.s. KLF8 KO-LM3 + MMP7i, *P* = 0.022). Consistently, western blotting showed that the content of cleaved Caspase3, an indicator of cell apoptosis, was significantly increased by KLF8-knockout in HCC, whereas knockdown of HMGA2 or MMP7 reversed KLF8^KO^-mediated Caspase3 activation (LM3 + Ctrli v.s. LM3 + HMGA2i, *P* < 0.001; KLF8 KO-LM3 + Ctrli v.s. KLF8 KO-LM3 + HMGA2i, *P* = 0.037; and LM3 + Ctrli v.s. LM3 + MMP7i, *P* = 0.011; KLF8 KO-LM3 + Ctrli v.s. KLF8 KO-LM3 + MMP7i, *P* = 0.010) (Fig. 5E, F). In short, these results demonstrated that both HMGA2 and MMP7 were critical in mediating the KLF8-induced anti-apoptotic effect in HCC, thus may providing potential therapeutic targets for HCC treatments.

## Discussion

As an important oncogene, KLF8 has drawn a lot of attention in the field of malignant tumors including HCC in recent years. However, a systematic analysis of molecules regulated by KLF8 was lacked. In this study, we used large-scale RNA-seq combined with ChIP-seq to conduct an in-depth analysis of functional genes regulated by KLF8 in HCC. With this combined approach, we found that KLF8 was mainly involved in cell apoptosis through directly regulating apoptotic-related genes. Moreover, we also identified novel pro-apoptotic genes in HCC, showing the application value of this sequencing strategy.

KLF8 was firstly identified as a CACCC-box binding protein that associates with CtBP and represses transcription [27]. The classical regulation mechanism of KLF8 was as a downstream target of focal adhesion kinase and an upstream regulator of cyclin D1 [28]. Besides, KLF8 was also found inducing epithelial to mesenchymal transition and epithelial cell invasion via directly bound and repressed the promoter of E-cadherin [11]. Poly A polymerase-1 was recognized as a KLF8-interacting and -regulating protein [29]. A reciprocal crosstalk between KLF8 and Wnt signal pathway was proposed in our previous work [12]. However, these fragmented findings make the role of KLF8 quite complicated in a certain situation. We aimed to investigate a novel functional role of KLF8 in regulating apoptosis of HCC, and fortunately the major role was quite obvious owing to the advanced methods. This strategy may help to achieve an in-depth and comprehensive understanding of a certain molecule in defined conditions, which is very important for a potential target.

HMGA2 is a member of the HMGA family which is an important component of DNA architecture [30, 31]. HMGA2 participates in many biological processes including the apoptotic pathway [32]. HMGA2 has been found overexpressed in many malignant tissues and is often considered as an oncogene [33–35], but few studies focused on the role of HMGA2 in HCC except for ones considering it as an oncogene on assumptions [36–38]. Our work shed a light on the complicated roles of HMGA2 in HCC. MMP7 is a secreted protease and a complicated participant in apoptosis. It has been reported inhibiting cell death in colon cancer cells while promoting smooth muscle cell apoptosis [26, 39]. Herein, MMP7 has been shown to promote cell apoptosis in HCC, but the detailed mechanism remains unclear. More work is warranted to make these two apoptotic-related genes as qualified targets for HCC treatments.

Although most of the transcription factors are commonly recognized as direct transcriptional activators, mounting evidence has indicated the key role of transcription factors in inducing epigenetic changes in specific regions of the genome [40, 41]. Specifically, posttranslational modifications of histones represent long-term regulatory effect and are tightly linked to epigenetic regulation of gene expression [42]. It is well established that aberrant H3K27ac in DNA regulatory regions were participated in the pathogenesis of various diseases, and previous large-scale studies also confirmed the correlation between alternations of H3K27ac and gene expression [43]. Consistent with this, our present study revealed that the genome-wide H3K27ac profiles were of great importance for better understanding of whole RNA expression alternations. Furthermore, by combined sequencing strategy and motif analysis, we associated epigenetic marks with functional gene expression patterns, and identified enrichment of sequence motifs in H3K27ac regions exhibiting alternations in response to cell apoptosis after KLF8 knockout. This led to the identification of potential target genes, such as HMGA2 and MMP7, which are critical for cell apoptosis. Our in vitro data was consistent with such sequencing findings. Suppression of HMGA2 or MMP7 by siRNA significantly resulted in blunted apoptosis response in HCC. Therefore, our findings of increased H3K27ac regions in apoptosis pathway-specific genes could be responsible, at least in part, for apoptosis-promoting effect on HCC cells.

There are several limitations in our study. First, the quality of anti-KLF8 ChIP-seq data was poor due to the low efficacy of KLF8 antibody, and the promoters regulated by KLF8 were analyzed by H3K27ac and motif analysis indirectly. This deficiency is expected to be compensated in the future. Second, although we identified HMGA2 and MMP7 as novel targets that involved in KLF8-meidiated anti-apoptosis effect in HCC, the underlying mechanisms have not yet been clearly elucidated, which requires further fundamental researches.

## Conclusions

In summary, our work offered a panoramic view of KLF8′s role in HCC for the first time, and found that KLF8 mainly regulated apoptotic-related genes directly. Meanwhile, our data also indicate a critical role of H3K27ac as an additional layer involved in the regulation of gene expression. Together, our findings confirm that the combined sequence approach may provide a useful strategy to establish an in-depth insight of KLF8′s role in HCC and thus facilitates the discovery of novel targets for HCC treatment.

## Supplementary information

**Additional file 1: Table S1.** Genes differentially expressed as well as H3K27 acetylation changed between KLF8^KO^-LM3 and LM3 cells.

**Additional file 2: Table S2.** Primers used for ChIP-quantitative real-time PCR.

**Additional file 3: Figure S1.** Expressions of KLF8 protein in the indicated cell lines were examined by Western blot assay. (A) Western blot analysis of KLF8 in KLF8^KO^-LM3 or LM3 cells using an anti-KLF8 antibody. (B) Western blot analysis of KLF8 in Huh-7 cells transfecting Flag-tagged KLF8 overexpressing lentivirus or control lentivirus using an anti-flag antibody. GAPDH was used as a loading control. **Figure S2.** Proliferation of the indicated cell lines was evaluated by determining the cell viability with Cell Counting Kit 8 Assay according to manufacturer’s instructions. The KLF8^OE^ stably transfected Huh-7 cell line and its negative control cell line (Ctrl^OE^) were established using lenti-virus system. The KLF8KO LM3 cell line was established using CRISPR/Cas9 technique.

## Data Availability

The datasets used and/or analysed during the current study are available from the corresponding author on reasonable request.

## Abbreviations

HCC: Hepatocellular carcinoma
KLF8: Krüppel-like factor 8
RNA-seq: RNA sequencing
ChIP: Chromatin immunoprecipitation
H3K27ac: Histone H3 lysine 27 acetylation
siRNA: Small interfering RNA
HMGA2: High-mobility group AT-hook 2
MMP7: Matrix metalloproteinase 7
DMEM: Dulbecco’s modified Eagle medium
FBS: Fetal Bovine Serum
CRISPR: Clustered regularly interspaced short palindromic repeats
GO: Gene Ontology
KEGG: Kyoto Encyclopedia of Genes and Genomes
SD: Standard deviation
