# Supplementary material for: A novel role of Krüppel-like factor 8 as an apoptosis repressor in hepatocellular carcinoma
Source: Cancer Cell Int. 2020 Aug 28;20:422. doi: 10.1186/s12935-020-01513-3 (PMC7456055; doi:10.1186/s12935-020-01513-3)
Supplement: Supplementary file 1 — Additional file 1: Table S1. Genes differentially expressed as well as H3K27 acetylation changed between KLF8KO-LM3 and LM3 cells. [file 12935_2020_1513_MOESM1_ESM.docx]

| **Table S1. Genes differentially expressed as well as H3K27 acetylation changed between KLF8KO-LM3 and LM3 cells** | | | | | | | | | | | | | | | | | | |  |
| --- | --- | --- | --- | --- | --- | --- | --- | --- | --- | --- | --- | --- | --- | --- | --- | --- | --- | --- | --- |
| **Chrome** | | **Start** | | **End** | | **NCBI** | | ***P*-value*** | | **Strand** | | **Gene** | | **Peak Scores** | | **Cell Death** | | **Motif Counts** | |
| chr8 | 122625270 | | 122653630 | | NM_005328 | | 1.79E-05 | | - | | *HAS2* | | 0.191283243339,0.599295787846,0.180865792617,0.0463284053282 | | no | | 1 | |  |
| chr7 | 89783688 | | 89794141 | | NM_012449 | | 1.89E-05 | | + | | *STEAP1* | | 0.227637688384,0.435178059266,0.326279794623 | | no | | 9 | |  |
| chr19 | 42259427 | | 42276113 | | NM_002483 | | 2.00E-05 | | + | | *CEACAM6* | | 0.426560956 | | no | | 11 | |  |
| chr7 | 16832263 | | 16844738 | | NM_006408 | | 2.16E-05 | | - | | *AGR2* | | 0.018097164689,0.526238893077 | | yes | | 4 | |  |
| chr2 | 234601511 | | 234681951 | | NM_001072 | | 2.33E-05 | | + | | *UGT1A6* | | 0.598098394 | | no | | 5 | |  |
| chr8 | 134249413 | | 134309547 | | NM_006096 | | 3.11E-05 | | - | | *NDRG1* | | 0.0150858207424,0.289963565529,0.23223627473,0.0453203101178 | | no | | 4 | |  |
| chr8 | 105501458 | | 105601252 | | NM_001135703 | | 3.85E-05 | | - | | *LRP12* | | 0.506616992366,0.13696908987,0.0371794161555 | | no | | 0 | |  |
| chr10 | 114135955 | | 114188138 | | NM_016234 | | 4.77E-05 | | + | | *ACSL5* | | 0.491644197 | | yes | | 11 | |  |
| chr1 | 40506254 | | 40538321 | | NM_006367 | | 6.54E-05 | | + | | *CAP1* | | 0.121237966433,0.309127953129,0.275821875565 | | no | | 12 | |  |
| chr11 | 58294343 | | 58345639 | | NM_001143995 | | 6.81E-05 | | - | | *LPXN* | | 0.146900469464,0.377569734576,0.0438930186509 | | no | | 4 | |  |
| chr20 | 18488209 | | 18542059 | | NM_001172746 | | 7.41E-05 | | + | | *SEC23B* | | 0.292877747811,0.528348064179,0.186001600587 | | no | | 5 | |  |
| chr4 | 7755816 | | 7780654 | | NR_026892 | | 9.55E-05 | | + | | *AFAP1-AS1* | | 0.108609108825,0.246596963942,0.459323740751 | | no | | 8 | |  |
| chr11 | 69924407 | | 70035652 | | NM_018043 | | 1.04E-04 | | + | | *ANO1* | | 0.231309185 | | no | | 17 | |  |
| chr1 | 162824086 | | 162838605 | | NM_178550 | | 1.09E-04 | | - | | *C1orf110* | | 0.454753393 | | no | | 0 | |  |
| chr11 | 118967212 | | 118972785 | | NM_001382 | | 1.14E-04 | | - | | *DPAGT1* | | 0.241714016897,0.546620774195,0.208879029669 | | no | | 8 | |  |
| chr3 | 42642146 | | 42690233 | | NM_005385 | | 1.18E-04 | | + | | *NKTR* | | 0.210556764 | | no | | 9 | |  |
| chr12 | 49315741 | | 49318664 | | NM_001143781 | | 1.31E-04 | | - | | *FKBP11* | | 0.55766324632,0.260279298379 | | no | | 5 | |  |
| chr17 | 30819539 | | 31204191 | | NM_015194 | | 1.46E-04 | | - | | *MYO1D* | | 0.365678773 | | no | | 9 | |  |
| chr7 | 134233848 | | 134264592 | | NM_001080538 | | 1.46E-04 | | + | | *AKR1B15* | | 0.0120824656162,0.0320646853279,0.0672055127397,0.476160641282 | | no | | 4 | |  |
| chr17 | 48351787 | | 48358846 | | NM_153229 | | 1.48E-04 | | + | | *TMEM92* | | 0.0578443208748,0.0150255979853 | | no | | 9 | |  |
| chr20 | 24986865 | | 25038818 | | NM_001252675 | | 1.48E-04 | | - | | *ACSS1* | | 0.135335283 | | no | | 10 | |  |
| chr1 | 8921058 | | 8939151 | | NM_001428 | | 1.52E-04 | | - | | *ENO1* | | 0.149269780922,0.593332695123,0.16762934781,0.0209419699926 | | no | | 10 | |  |
| chr7 | 1473994 | | 1499109 | | NM_182924 | | 1.67E-04 | | - | | *MICALL2* | | 0.136149735851,0.348540793888,0.151677306259,0.0478348894942, | | no | | 19 | |  |
|  |  | |  | |  | |  | |  | |  | | 0.0138426620865 | |  | |  | |  |
| chr7 | 29523431 | | 29553951 | | NM_001293081 | | 1.70E-04 | | + | | *CHN2* | | 0.189001561888,0.582748252374 | | no | | 2 | |  |
| chr2 | 238232654 | | 238322850 | | NM_004369 | | 1.73E-04 | | - | | *COL6A3* | | 0.196321825 | | no | | 12 | |  |
| chr2 | 43864438 | | 43995126 | | NM_172069 | | 1.75E-04 | | + | | *PLEKHH2* | | 0.032257652 | | no | | 6 | |  |
| chr4 | 74861358 | | 74864446 | | NM_002994 | | 1.77E-04 | | - | | *CXCL5* | | 0.342323186 | | no | | 3 | |  |
| chr12 | 6421816 | | 6437672 | | NM_001144857 | | 1.87E-04 | | + | | *PLEKHG6* | | 0.0277089448263,0.330218738555,0.373812852945,0.117654843022 | | no | | 16 | |  |
| chr19 | 10501808 | | 10514271 | | NM_007065 | | 1.91E-04 | | - | | *CDC37* | | 0.12051271656,0.321100859871,0.320459299925 | | no | | 12 | |  |
| chr11 | 64008603 | | 64011607 | | NM_001135208 | | 1.99E-04 | | + | | *FKBP2* | | 0.244632058332,0.585083913592 | | no | | 19 | |  |
| chr7 | 33053724 | | 33080777 | | NM_001166118 | | 2.01E-04 | | - | | *NT5C3A* | | 0.0616673907962,0.198691879183,0.532591801007,0.0824139961748 | | no | | 3 | |  |
| chr1 | 113162074 | | 113214241 | | NM_006135 | | 2.12E-04 | | + | | *CAPZA1* | | 0.522045776761,0.166960169667 | | no | | 9 | |  |
| chr12 | 66218239 | | 66360071 | | NM_003483 | | 2.15E-04 | | + | | *HMGA2* | | 0.194368391 | | yes | | 15 | |  |
| chr19 | 54960339 | | 54973226 | | NM_052925 | | 2.17E-04 | | + | | *LENG8* | | 0.195538106577,0.267135301966 | | no | | 9 | |  |
| chr2 | 191745546 | | 191830270 | | NM_014905 | | 2.18E-04 | | + | | *GLS* | | 0.280270519824,0.260800377881,0.063545442443,0.0124504314756 | | no | | 10 | |  |
| chr4 | 38968365 | | 39034041 | | NM_001303228 | | 2.45E-04 | | - | | *TMEM156* | | 0.0142357339506,0.110140329886,0.343695220928,0.349238573022 | | no | | 8 | |  |
| chr4 | 74437266 | | 74486287 | | NM_177532 | | 2.50E-04 | | - | | *RASSF6* | | 0.514788058 | | yes | | 1 | |  |
| chr1 | 63906440 | | 63988944 | | NM_001206739 | | 2.58E-04 | | - | | *ITGB3BP* | | 0.495593125693,0.211400677654 | | yes | | 2 | |  |
| chr4 | 95373007 | | 95589378 | | NM_006457 | | 2.63E-04 | | + | | *PDLIM5* | | 0.149269780922,0.0448693654976 | | no | | 12 | |  |
| chr7 | 95401817 | | 95727736 | | NM_004411 | | 2.65E-04 | | + | | *DYNC1I1* | | 0.546620774195,0.250073601112 | | no | | 10 | |  |
| chrY | 15017615 | | 15030439 | | NM_001302552 | | 2.76E-04 | | + | | *DDX3Y* | | 0.0562471447042,0.245121812039,0.388291085606 | | no | | 5 | |  |
| chr8 | 87111138 | | 87166454 | | NM_152565 | | 2.78E-04 | | + | | *ATP6V0D2* | | 0.463013068311,0.15755195533 | | no | | 5 | |  |
| chr2 | 224839764 | | 224904036 | | NM_001136528 | | 2.79E-04 | | - | | *SERPINE2* | | 0.258205370763,0.2785939315 | | no | | 2 | |  |
| chr1 | 6052357 | | 6161253 | | NM_001199861 | | 2.89E-04 | | + | | *KCNAB2* | | 0.573498475876,0.0350843541008 | | no | | 14 | |  |
| chr3 | 65344943 | | 66024509 | | NM_004742 | | 2.96E-04 | | - | | *MAGI1* | | 0.206800648598,0.444858066223,0.182318522128 | | yes | | 3 | |  |
| chr20 | 33759739 | | 33765165 | | NM_006404 | | 2.98E-04 | | + | | *PROCR* | | 0.315372752 | | no | | 4 | |  |
| chr10 | 14560555 | | 14614372 | | NM_001282700 | | 2.99E-04 | | - | | *FAM107B* | | 0.0146107166851,0.117419768489,0.477113915521,0.0998586093503, | | no | | 1 | |  |
|  |  | |  | |  | |  | |  | |  | | 0.0151766077556 | |  | |  | |  |
| chr1 | 24286300 | | 24289949 | | NM_017761 | | 3.00E-04 | | + | | *PNRC2* | | 0.309127953129,0.401719980098 | | no | | 5 | |  |
| chr1 | 59120410 | | 59165747 | | NM_001085487 | | 3.03E-04 | | - | | *MYSM1* | | 0.392193476 | | no | | 4 | |  |
| chr8 | 144640476 | | 144645231 | | NM_024736 | | 3.12E-04 | | + | | *GSDMD* | | 0.372320588053,0.393765391532 | | no | | 16 | |  |
| chr5 | 73923230 | | 73937249 | | NM_001256574 | | 3.27E-04 | | - | | *ENC1* | | 0.04688769522,0.316004128692,0.273077402047,0.0360085092644 | | no | | 3 | |  |
| chr22 | 39130718 | | 39151467 | | NM_001199580 | | 3.33E-04 | | - | | *SUN2* | | 0.0577287478446,0.163981772603,0.379841962851,0.295230166924 | | no | | 13 | |  |
| chr18 | 74724738 | | 74844774 | | NM_001025100 | | 3.52E-04 | | - | | *MBP* | | 0.0787088246188,0.307893911046,0.336216493707 | | no | | 6 | |  |
| chr19 | 41099071 | | 41135725 | | NM_003573 | | 3.58E-04 | | + | | *LTBP4* | | 0.027820003 | | no | | 8 | |  |
| chr21 | 43782390 | | 43786644 | | NM_003225 | | 3.66E-04 | | - | | *TFF1* | | 0.551011278991,0.140576985608 | | no | | 5 | |  |
| chr12 | 30862485 | | 30907448 | | NM_023925 | | 3.66E-04 | | - | | *CAPRIN2* | | 0.1062458 | | no | | 3 | |  |
| chr7 | 22602955 | | 22613617 | | NR_038393 | | 3.71E-04 | | + | | *LOC100506178* | | 0.254615682 | | no | | 9 | |  |
| chr1 | 75171171 | | 75199092 | | NM_001130043 | | 4.12E-04 | | - | | *CRYZ* | | 0.570067787 | | no | | 2 | |  |
| chr17 | 62774256 | | 62777622 | | NR_027487 | | 4.17E-04 | | - | | *LOC146880* | | 0.0335070303827,0.138069237311 | | no | | 0 | |  |
| chr1 | 200520624 | | 200589862 | | NM_014875 | | 4.29E-04 | | - | | *KIF14* | | 0.476160641 | | yes | | 5 | |  |
| chr1 | 87170252 | | 87213867 | | NM_001206653 | | 4.36E-04 | | + | | *SH3GLB1* | | 0.0536110540442,0.193980042291,0.528348064179 | | yes | | 10 | |  |
| chr18 | 29202208 | | 29264686 | | NM_004775 | | 4.56E-04 | | - | | *B4GALT6* | | 0.265537288987,0.182683524053 | | no | | 3 | |  |
| chrX | 102930425 | | 102941746 | | NM_001142430 | | 4.63E-04 | | - | | *MORF4L2* | | 0.119791805158,0.323033256422,0.211400677654,0.074422274017 | | no | | 4 | |  |
| chrX | 122734411 | | 122866904 | | NM_001081550 | | 4.66E-04 | | - | | *THOC2* | | 0.414782911682,0.349238573022 | | no | | 4 | |  |
| chr20 | 2633177 | | 2639039 | | NR_027700 | | 4.68E-04 | | + | | *NOP56* | | 0.334874313881,0.339595525645 | | no | | 8 | |  |
| chr6 | 112429133 | | 112575917 | | NM_002290 | | 4.69E-04 | | - | | *LAMA4* | | 0.269280956 | | no | | 5 | |  |
| chr3 | 186507681 | | 186524484 | | NM_181573 | | 4.73E-04 | | - | | *RFC4* | | 0.279151676922,0.542265253314 | | no | | 2 | |  |
| chr3 | 69024362 | | 69063045 | | NM_173654 | | 4.74E-04 | | - | | *EOGT* | | 0.292877747811,0.50560477094 | | no | | 6 | |  |
| chr17 | 7123149 | | 7128586 | | NM_000018 | | 4.77E-04 | | + | | *ACADVL* | | 0.163654137 | | no | | 15 | |  |
| chr22 | 24891250 | | 24922553 | | NM_016327 | | 4.80E-04 | | + | | *UPB1* | | 0.271987274 | | no | | 13 | |  |
| chr11 | 102391238 | | 102401478 | | NM_002423 | | 4.86E-04 | | - | | *MMP7* | | 0.483840486 | | yes | | 7 | |  |
| chr8 | 86157715 | | 86196302 | | NM_198584 | | 5.06E-04 | | + | | *CA13* | | 0.571209063849,0.207629507805 | | no | | 8 | |  |
| chr2 | 27193238 | | 27250087 | | NM_012326 | | 5.07E-04 | | + | | *MAPRE3* | | 0.0234707573684,0.334205234556,0.07607770704 | | no | | 2 | |  |
| chr7 | 99156447 | | 99162328 | | NM_001085367 | | 5.09E-04 | | + | | *ZNF655* | | 0.590964105 | | no | | 7 | |  |
| chr3 | 170780291 | | 171178197 | | NM_001161566 | | 5.09E-04 | | - | | *TNIK* | | 0.359874471002,0.113721818647 | | yes | | 3 | |  |
| chr1 | 38275238 | | 38325292 | | NM_005955 | | 5.16E-04 | | - | | *MTF1* | | 0.345072755043,0.271443843212,0.0912638972031 | | no | | 8 | |  |
| chr8 | 93895757 | | 93977817 | | NM_001191036 | | 5.34E-04 | | - | | *TRIQK* | | 0.120753983179,0.358437848279,0.298794270781 | | no | | 3 | |  |
| chr16 | 64980682 | | 65155919 | | NM_001797 | | 5.41E-04 | | - | | *CDH11* | | 0.044600955 | | no | | 5 | |  |
| chr3 | 42589458 | | 42623520 | | NM_001201584 | | 5.42E-04 | | - | | *SEC22C* | | 0.345072755043,0.400917343042 | | no | | 6 | |  |
| chr1 | 94883932 | | 94984219 | | NM_002858 | | 5.45E-04 | | + | | *ABCD3* | | 0.28593236 | | no | | 13 | |  |
| chr1 | 32117847 | | 32169768 | | NM_001856 | | 5.53E-04 | | - | | *COL16A1* | | 0.099659092 | | no | | 4 | |  |
| chr2 | 54952148 | | 55199156 | | NM_001039753 | | 5.85E-04 | | + | | *EML6* | | 0.011986192 | | no | | 12 | |  |
| chr12 | 56510373 | | 56511616 | | NM_001035267 | | 5.88E-04 | | + | | *RPL41* | | 0.254615682025,0.327587527524,0.139456856215 | | no | | 2 | |  |
| chr1 | 45205489 | | 45233438 | | NM_006845 | | 5.93E-04 | | + | | *KIF2C* | | 0.347844409 | | no | | 3 | |  |
| chr1 | 93646272 | | 93744287 | | NM_206886 | | 5.95E-04 | | + | | *CCDC18* | | 0.219149748442,0.427414931949 | | no | | 7 | |  |
| chr3 | 52719935 | | 52728510 | | NM_014366 | | 5.95E-04 | | + | | *GNL3* | | 0.500573919412,0.251075898775 | | no | | 3 | |  |
| chr9 | 114312001 | | 114362135 | | NM_001146109 | | 6.00E-04 | | - | | *PTGR1* | | 0.327587527524,0.307893911046,0.0441571684197 | | no | | 3 | |  |
| chr1 | 84335056 | | 84464833 | | NM_024686 | | 6.02E-04 | | - | | *TTLL7* | | 0.448431204 | | no | | 1 | |  |
| chr20 | 44441214 | | 44445596 | | NM_181800 | | 6.15E-04 | | + | | *UBE2C* | | 0.246104262879,0.372320588053 | | no | | 13 | |  |
| chr17 | 25621105 | | 25640645 | | NM_015626 | | 6.17E-04 | | + | | *WSB1* | | 0.227637688384,0.423162082318 | | no | | 9 | |  |
| chr19 | 38874991 | | 38878668 | | NM_152657 | | 6.31E-04 | | - | | *GGN* | | 0.458406011305,0.287078379846,0.0550232200564 | | no | | 13 | |  |
| chr2 | 235401685 | | 235405693 | | NM_005737 | | 6.33E-04 | | - | | *ARL4C* | | 0.0599646534212,0.433440823817 | | no | | 4 | |  |
| chr17 | 16318855 | | 16340317 | | NM_016113 | | 6.40E-04 | | + | | *TRPV2* | | 0.215240343171,0.0586598366575 | | no | | 15 | |  |
| chr7 | 97920961 | | 98030427 | | NM_018842 | | 6.45E-04 | | - | | *BAIAP2L1* | | 0.014965615638,0.353454681959,0.268205983108 | | no | | 6 | |  |
| chr1 | 52254865 | | 52344609 | | NM_001101662 | | 6.47E-04 | | - | | *NRD1* | | 0.237877367669,0.275821875565 | | no | | 0 | |  |
| chr7 | 36363758 | | 36429734 | | NM_001100425 | | 6.51E-04 | | - | | *KIAA0895* | | 0.5957107789,0.14660696213,0.0253747676072 | | no | | 4 | |  |
| chr3 | 86987122 | | 87040257 | | NM_016206 | | 6.67E-04 | | - | | *VGLL3* | | 0.210136071 | | no | | 6 | |  |
| chr7 | 111366163 | | 111846462 | | NM_014705 | | 6.67E-04 | | - | | *DOCK4* | | 0.0728028628274,0.217838786769,0.22135224003 | | no | | 2 | |  |
| chr19 | 44152722 | | 44174498 | | NM_002659 | | 6.75E-04 | | - | | *PLAUR* | | 0.059367995 | | yes | | 9 | |  |
| chr1 | 42922011 | | 42926901 | | NM_001287506 | | 6.86E-04 | | + | | *PPCS* | | 0.415613307624,0.271443843212 | | no | | 7 | |  |
| chr12 | 8234806 | | 8250373 | | NR_024260 | | 6.87E-04 | | + | | *NECAP1* | | 0.385197149168,0.382127865479 | | no | | 3 | |  |
| chr17 | 38249036 | | 38256978 | | NM_021724 | | 6.90E-04 | | - | | *NR1D1* | | 0.321100859871,0.0834089217433 | | no | | 16 | |  |
| chr1 | 43391045 | | 43424847 | | NM_006516 | | 6.95E-04 | | - | | *SLC2A1* | | 0.216535667 | | no | | 14 | |  |
| chr1 | 6526151 | | 6551761 | | NM_020631 | | 7.07E-04 | | - | | *PLEKHG5* | | 0.208879029669,0.231309185046 | | yes | | 13 | |  |
| chr1 | 114437370 | | 114447525 | | NM_001253852 | | 7.21E-04 | | - | | *AP4B1* | | 0.383659438 | | no | | 2 | |  |
| chr19 | 42702744 | | 42724304 | | NM_001270614 | | 7.33E-04 | | - | | *DEDD2* | | 0.0146107166851,0.318542300873,0.307278738601 | | yes | | 17 | |  |
| chr17 | 26904582 | | 26926056 | | NM_006461 | | 7.35E-04 | | - | | *SPAG5* | | 0.3693539059,0.294640296657,0.0586598366575 | | no | | 15 | |  |
| chr12 | 56710006 | | 56727837 | | NM_001166279 | | 7.36E-04 | | - | | *PAN2* | | 0.407383612742,0.322387835545 | | no | | 6 | |  |
| chr6 | 49518112 | | 49519808 | | NM_001145652 | | 7.47E-04 | | + | | *C6orf141* | | 0.171701118 | | no | | 6 | |  |
| chr18 | 21111462 | | 21166581 | | NM_000271 | | 7.51E-04 | | - | | *NPC1* | | 0.117654843022,0.545528625159,0.106885191201 | | no | | 5 | |  |
| chr12 | 31226778 | | 31257725 | | NM_004399 | | 7.54E-04 | | + | | *DDX11* | | 0.461164715209,0.180865792617 | | no | | 11 | |  |
| chr1 | 45477804 | | 45481341 | | NM_000374 | | 7.63E-04 | | + | | *UROD* | | 0.30300681 | | no | | 11 | |  |
| chr9 | 129849627 | | 129885044 | | NM_012098 | | 7.71E-04 | | - | | *ANGPTL2* | | 0.372320588 | | no | | 11 | |  |
| chr1 | 22004791 | | 22109688 | | NM_032236 | | 7.71E-04 | | - | | *USP48* | | 0.373812853 | | no | | 4 | |  |
| chr1 | 24018268 | | 24022915 | | NM_001199802 | | 7.86E-04 | | + | | *RPL11* | | 0.390627835 | | no | | 5 | |  |
| chr2 | 203130514 | | 203168384 | | NM_015934 | | 7.86E-04 | | + | | *NOP58* | | 0.308510315066,0.408199195278 | | no | | 6 | |  |
| chr1 | 33789223 | | 33815499 | | NM_004427 | | 7.87E-04 | | - | | *PHC2* | | 0.239788019 | | no | | 12 | |  |
| chr1 | 38032412 | | 38061586 | | NM_013285 | | 7.89E-04 | | - | | *GNL2* | | 0.329558961 | | no | | 7 | |  |
| chr1 | 150190716 | | 150208504 | | NM_001136478 | | 7.90E-04 | | - | | *ANP32E* | | 0.201090545 | | no | | 1 | |  |
| chr7 | 36429411 | | 36493401 | | NM_001284302 | | 8.14E-04 | | + | | *ANLN* | | 0.323679969433,0.0768423007072,0.0132998835424 | | no | | 8 | |  |
| chr7 | 937536 | | 966852 | | NM_001284310 | | 8.18E-04 | | - | | *ADAP1* | | 0.0440689423383,0.478069098216,0.0832422706065, | | no | | 13 | |  |
|  |  | |  | |  | |  | |  | |  | | 0.0410075123916,0.0126765683685 | |  | |  | |  |
| chr7 | 7222245 | | 7288280 | | NM_020156 | | 8.19E-04 | | + | | *C1GALT1* | | 0.194368390594,0.422316603913 | | no | | 7 | |  |
| chr8 | 121547984 | | 121824309 | | NM_021021 | | 8.28E-04 | | - | | *SNTB1* | | 0.026516184 | | no | | 3 | |  |
| chr1 | 155882833 | | 155904233 | | NM_014949 | | 8.31E-04 | | - | | *KIAA0907* | | 0.271443843212,0.245121812039 | | no | | 0 | |  |
| chr1 | 46769284 | | 46782449 | | NM_001297566 | | 8.37E-04 | | + | | *UQCRH* | | 0.288806028 | | no | | 9 | |  |
| chr1 | 94994731 | | 95007413 | | NM_001993 | | 8.39E-04 | | - | | *F3* | | 0.25410696 | | yes | | 3 | |  |
| chr17 | 33762114 | | 33775856 | | NM_144682 | | 8.40E-04 | | - | | *SLFN13* | | 0.370093352912,0.356293660186 | | no | | 6 | |  |
| chr1 | 93615298 | | 93646246 | | NM_001167830 | | 8.45E-04 | | - | | *TMED5* | | 0.230847028986,0.405757333019 | | no | | 4 | |  |
| chr2 | 33701320 | | 33789798 | | NM_001139488 | | 8.54E-04 | | + | | *RASGRP3* | | 0.015116023 | | no | | 4 | |  |
| chr9 | 75766646 | | 75785310 | | NM_000700 | | 8.59E-04 | | + | | *ANXA1* | | 0.035721590396,0.32174370422,0.22809341934 | | yes | | 2 | |  |
| chr3 | 185677757 | | 185698665 | | NR_033752 | | 8.68E-04 | | + | | *LOC344887* | | 0.0654806892775,0.198691879183,0.212672894553 | | no | | 0 | |  |
| chr20 | 48807119 | | 48809227 | | NM_001285878 | | 8.71E-04 | | + | | *CEBPB* | | 0.040762204 | | yes | | 20 | |  |
| chr1 | 151032150 | | 151040973 | | NM_006818 | | 8.80E-04 | | + | | *MLLT11* | | 0.0259911287788,0.073094657479,0.310366941265 | | yes | | 9 | |  |
| chr1 | 94335013 | | 94344762 | | NM_014597 | | 8.82E-04 | | - | | *DNTTIP2* | | 0.0573834123999,0.362040208543 | | no | | 5 | |  |
| chrX | 153639853 | | 153650063 | | NM_000116 | | 9.03E-04 | | + | | *TAZ* | | 0.0195652548029,0.395343607426,0.325627887159 | | no | | 17 | |  |
| chr1 | 43148065 | | 43168020 | | NM_004559 | | 9.11E-04 | | + | | *YBX1* | | 0.281957198 | | no | | 10 | |  |
| chr2 | 220363586 | | 220371718 | | NM_013335 | | 9.13E-04 | | + | | *GMPPA* | | 0.182318522 | | no | | 11 | |  |
| chr12 | 51442081 | | 51454207 | | NR_045020 | | 9.32E-04 | | + | | *LETMD1* | | 0.360594940173,0.347844408917 | | no | | 6 | |  |
| chr1 | 26856248 | | 26901520 | | NM_002953 | | 9.33E-04 | | + | | *RPS6KA1* | | 0.014581524 | | yes | | 17 | |  |
| chr2 | 55459038 | | 55462989 | | NM_001135592 | | 9.38E-04 | | + | | *RPS27A* | | 0.495593125693,0.075320721207 | | yes | | 8 | |  |
| chr9 | 33750818 | | 33799229 | | NM_001197098 | | 9.40E-04 | | + | | *PRSS3* | | 0.116251422271,0.0113107689801 | | no | | 12 | |  |
| chr7 | 89841173 | | 89866992 | | NM_001244946 | | 9.43E-04 | | + | | *STEAP2* | | 0.174819721307,0.46579949765 | | no | | 10 | |  |
| chr1 | 70671364 | | 70717701 | | NM_004768 | | 9.58E-04 | | + | | *SRSF11* | | 0.413127094 | | no | | 2 | |  |
| chr1 | 40723721 | | 40759856 | | NM_005857 | | 9.75E-04 | | + | | *ZMPSTE24* | | 0.305440586141,0.262369883958 | | no | | 4 | |  |
| chr7 | 90032647 | | 90045268 | | NM_001185073 | | 9.76E-04 | | + | | *CLDN12* | | 0.342323185944,0.0527601029419,0.0131675474901 | | no | | 2 | |  |
| chr20 | 16710608 | | 16722417 | | NM_003092 | | 9.80E-04 | | + | | *SNRPB2* | | 0.310988296296,0.360594940173 | | no | | 8 | |  |
| chr8 | 145734418 | | 145736611 | | NR_130120 | | 9.84E-04 | | + | | *MFSD3* | | 0.239788019025,0.336889599576 | | no | | 19 | |  |
| chr16 | 50300450 | | 50344440 | | NM_001286057 | | 9.98E-04 | | + | | *ADCY7* | | 0.0122038964144,0.0829099665752,0.236454376705,0.354162298704 | | no | | 12 | |  |
| chr12 | 3000032 | | 3050306 | | NM_003324 | | 1.01E-03 | | + | | *TULP3* | | 0.270901498052,0.385197149168 | | no | | 8 | |  |
| chr1 | 53708040 | | 53793821 | | NM_033300 | | 1.02E-03 | | - | | *LRP8* | | 0.073534544 | | no | | 3 | |  |
| chr12 | 27677044 | | 27848497 | | NM_001198916 | | 1.02E-03 | | + | | *PPFIBP1* | | 0.313486180883,0.413954174871 | | no | | 15 | |  |
| chr7 | 32524944 | | 32530475 | | NR_024466 | | 1.02E-03 | | - | | *LSM5* | | 0.436049286322,0.292292577681 | | no | | 0 | |  |
| chr20 | 10618331 | | 10654694 | | NM_000214 | | 1.04E-03 | | - | | *JAG1* | | 0.0368831674012,0.173080236016,0.19908966059,0.0585426342257 | | no | | 5 | |  |
| chr1 | 33087306 | | 33116185 | | NM_178547 | | 1.05E-03 | | - | | *ZBTB8OS* | | 0.419790291 | | no | | 5 | |  |
| chr2 | 74652987 | | 74669060 | | NM_001015055 | | 1.06E-03 | | - | | *RTKN* | | 0.471422763739,0.0898152945722 | | yes | | 16 | |  |
| chr17 | 74708913 | | 74722881 | | NM_001081461 | | 1.06E-03 | | - | | *JMJD6* | | 0.220909977959,0.354162298704 | | no | | 9 | |  |
| chr3 | 10068112 | | 10141344 | | NM_033084 | | 1.06E-03 | | + | | *FANCD2* | | 0.319819021816,0.379083038103 | | no | | 2 | |  |
| chrX | 105937067 | | 106040246 | | NM_024539 | | 1.08E-03 | | + | | *RNF128* | | 0.0890996386562,0.541181806615,0.01560755792 | | no | | 1 | |  |
| chr1 | 46092975 | | 46152302 | | NM_021639 | | 1.09E-03 | | - | | *GPBP1L1* | | 0.364948146454,0.0713612695564 | | no | | 4 | |  |
| chr7 | 643034 | | 648139 | | NR_110055 | | 1.09E-03 | | + | | *LOC101926963* | | 0.109700648516,0.499573772054,0.0645703468932 | | no | | 27 | |  |
| chr19 | 45542297 | | 45574214 | | NM_007056 | | 1.09E-03 | | + | | *CLASRP* | | 0.443082188 | | no | | 10 | |  |
| chr17 | 46048321 | | 46059152 | | NM_176096 | | 1.10E-03 | | + | | *CDK5RAP3* | | 0.261845669 | | yes | | 8 | |  |
| chr1 | 52870218 | | 52883992 | | NM_032864 | | 1.11E-03 | | + | | *PRPF38A* | | 0.0783946181522,0.245612546234,0.374561226776 | | no | | 9 | |  |
| chr1 | 1634169 | | 1655791 | | NM_033529 | | 1.11E-03 | | - | | *CDK11A* | | 0.479985204 | | yes | | 20 | |  |
| chr17 | 2240805 | | 2284348 | | NM_014853 | | 1.13E-03 | | + | | *SGSM2* | | 0.047930655 | | no | | 12 | |  |
| chr1 | 895966 | | 901099 | | NM_198317 | | 1.14E-03 | | + | | *KLHL17* | | 0.026043163 | | no | | 17 | |  |
| chr11 | 58912251 | | 58922511 | | NM_022074 | | 1.14E-03 | | + | | *FAM111A* | | 0.309746827703,0.337564053003 | | no | | 9 | |  |
| chr5 | 35856976 | | 35879705 | | NM_002185 | | 1.15E-03 | | + | | *IL7R* | | 0.23316708 | | no | | 1 | |  |
| chr7 | 27779713 | | 27869386 | | NM_001079864 | | 1.17E-03 | | + | | *TAX1BP1* | | 0.313486180883,0.301797203126 | | yes | | 9 | |  |
| chr1 | 45241245 | | 45244412 | | NM_001012 | | 1.17E-03 | | + | | *RPS8* | | 0.0908995707529,0.277481781556 | | no | | 7 | |  |
| chr19 | 38755097 | | 38783254 | | NM_001166103 | | 1.17E-03 | | + | | *SPINT2* | | 0.375311099 | | no | | 10 | |  |
| chr11 | 118914895 | | 118927957 | | NM_006389 | | 1.17E-03 | | - | | *HYOU1* | | 0.091629684 | | yes | | 10 | |  |
| chr1 | 45794913 | | 45805788 | | NM_001293196 | | 1.19E-03 | | - | | *MUTYH* | | 0.170332988825,0.439551671473 | | no | | 17 | |  |
| chr1 | 101337927 | | 101360735 | | NM_001261440 | | 1.21E-03 | | - | | *EXTL2* | | 0.504594571934,0.0502874367236 | | no | | 4 | |  |
| chr8 | 144656954 | | 144660521 | | NM_001286829 | | 1.21E-03 | | - | | *NAPRT* | | 0.187120964925,0.0331736298598 | | no | | 28 | |  |
| chr3 | 186288464 | | 186303589 | | NM_016306 | | 1.21E-03 | | + | | *DNAJB11* | | 0.192434392794,0.462087967584 | | no | | 11 | |  |
| chr12 | 53342842 | | 53346685 | | NM_000224 | | 1.23E-03 | | + | | *KRT18* | | 0.261322500586,0.0506913497151 | | yes | | 13 | |  |
| chr7 | 75625654 | | 75677321 | | NM_016086 | | 1.23E-03 | | - | | *STYXL1* | | 0.326933007207,0.339595525645 | | no | | 11 | |  |
| chr17 | 53469973 | | 53499341 | | NM_012329 | | 1.24E-03 | | - | | *MMD* | | 0.077925659 | | no | | 3 | |  |
| chr1 | 47799468 | | 47844511 | | NM_016308 | | 1.24E-03 | | + | | *CMPK1* | | 0.15755195533,0.0339115369325 | | no | | 5 | |  |
| chr16 | 1756220 | | 1820318 | | NM_001040439 | | 1.26E-03 | | + | | *MAPK8IP3* | | 0.0894567509592,0.290544072974 | | no | | 13 | |  |
| chr2 | 200134222 | | 200322819 | | NM_001172509 | | 1.26E-03 | | - | | *SATB2* | | 0.018097164689,0.0331736298598 | | no | | 3 | |  |
| chr4 | 80822770 | | 80994477 | | NM_058172 | | 1.27E-03 | | - | | *ANTXR2* | | 0.310988296 | | no | | 9 | |  |
| chr3 | 38207025 | | 38296979 | | NM_005109 | | 1.28E-03 | | + | | *OXSR1* | | 0.063545442443,0.428270617213 | | yes | | 11 | |  |
| chr20 | 3869741 | | 3904502 | | NM_153638 | | 1.28E-03 | | + | | *PANK2* | | 0.456576049623,0.240268074959 | | no | | 8 | |  |
| chr8 | 145579087 | | 145582183 | | NM_012162 | | 1.28E-03 | | - | | *FBXL6* | | 0.359874471 | | no | | 18 | |  |
| chr1 | 21069170 | | 21113181 | | NM_016287 | | 1.28E-03 | | - | | *HP1BP3* | | 0.367144417558,0.262895148816 | | no | | 6 | |  |
| chr20 | 44420575 | | 44440066 | | NM_052951 | | 1.29E-03 | | + | | *DNTTIP1* | | 0.358437848279,0.359874471002 | | no | | 9 | |  |
| chr8 | 48920994 | | 48974454 | | NM_003350 | | 1.32E-03 | | + | | *UBE2V2* | | 0.562142445 | | yes | | 3 | |  |
| chr15 | 32907344 | | 32932150 | | NM_001286479 | | 1.33E-03 | | + | | *ARHGAP11A* | | 0.551011278991,0.157867374555 | | no | | 5 | |  |
| chr1 | 111728590 | | 111747160 | | NM_001271833 | | 1.34E-03 | | - | | *DENND2D* | | 0.400917343 | | no | | 7 | |  |
| chr4 | 75230859 | | 75254477 | | NM_001432 | | 1.34E-03 | | + | | *EREG* | | 0.51375951123,0.022914165016 | | no | | 4 | |  |
| chr1 | 42642209 | | 42800636 | | NM_001198851 | | 1.35E-03 | | - | | *FOXJ3* | | 0.110360830973,0.356293660186,0.162350126703 | | no | | 8 | |  |
| chr1 | 38478383 | | 38490497 | | NM_016037 | | 1.35E-03 | | + | | *UTP11L* | | 0.272531793 | | yes | | 0 | |  |
| chr20 | 5918478 | | 5931204 | | NM_001281467 | | 1.36E-03 | | - | | *TRMT6* | | 0.379841963 | | no | | 2 | |  |
| chr1 | 93775665 | | 93811368 | | NR_034089 | | 1.36E-03 | | - | | *LOC100131564* | | 0.163654137 | | no | | 0 | |  |
| chrX | 109437413 | | 109561380 | | NM_015365 | | 1.37E-03 | | - | | *AMMECR1* | | 0.347844409 | | no | | 4 | |  |
| chr17 | 7387697 | | 7417935 | | NM_000937 | | 1.37E-03 | | + | | *POLR2A* | | 0.168975761 | | no | | 12 | |  |
| chr10 | 127512103 | | 127542264 | | NM_016567 | | 1.37E-03 | | + | | *BCCIP* | | 0.477113916 | | no | | 9 | |  |
| chr7 | 6448746 | | 6487643 | | NM_139179 | | 1.39E-03 | | - | | *DAGLB* | | 0.260279298379,0.366410862522 | | no | | 6 | |  |
| chr7 | 43622691 | | 43666978 | | NM_004760 | | 1.39E-03 | | + | | *STK17A* | | 0.342323186 | | yes | | 6 | |  |
| chr11 | 124636393 | | 124670299 | | NM_001301087 | | 1.41E-03 | | - | | *MSANTD2* | | 0.354871332 | | no | | 0 | |  |
| chr1 | 28929608 | | 28969604 | | NM_001135218 | | 1.41E-03 | | - | | *TAF12* | | 0.275270783 | | no | | 3 | |  |
| chr1 | 89401455 | | 89458643 | | NM_001008661 | | 1.43E-03 | | - | | *CCBL2* | | 0.294640297 | | no | | 0 | |  |
| chr20 | 5931297 | | 5975852 | | NM_182802 | | 1.43E-03 | | + | | *MCM8* | | 0.315372752 | | no | | 7 | |  |
| chr19 | 50364459 | | 50370822 | | NM_007254 | | 1.44E-03 | | - | | *PNKP* | | 0.563267855 | | no | | 22 | |  |
| chr8 | 128902873 | | 129113499 | | NR_003367 | | 1.44E-03 | | + | | *PVT1* | | 0.341639223762,0.347149415325 | | no | | 3 | |  |
| chr16 | 2047652 | | 2059822 | | NM_178167 | | 1.45E-03 | | - | | *ZNF598* | | 0.333537492052,0.27417189919 | | no | | 13 | |  |
| chr3 | 142680073 | | 142682178 | | NM_198504 | | 1.45E-03 | | - | | *PAQR9* | | 0.348540794 | | no | | 5 | |  |
| chr20 | 10385427 | | 10414887 | | NR_072977 | | 1.45E-03 | | - | | *MKKS* | | 0.303613429576,0.155361596245,0.0420035979034 | | no | | 1 | |  |
| chr2 | 17721806 | | 17837706 | | NM_003385 | | 1.45E-03 | | + | | *VSNL1* | | 0.045959256649,0.316004128692 | | no | | 11 | |  |
| chr2 | 238600806 | | 238690290 | | NM_001137551 | | 1.46E-03 | | + | | *LRRFIP1* | | 0.163981772603,0.259759259994 | | no | | 17 | |  |
| chr8 | 74206836 | | 74237520 | | NM_172037 | | 1.46E-03 | | + | | *RDH10* | | 0.118362894109,0.127453969895,0.0329092995519 | | no | | 8 | |  |
| chr3 | 149036284 | | 149051259 | | NM_001184723 | | 1.46E-03 | | - | | *TM4SF18* | | 0.499573772 | | no | | 7 | |  |
| chr8 | 145637797 | | 145642279 | | NM_130849 | | 1.47E-03 | | - | | *SLC39A4* | | 0.568928791 | | no | | 16 | |  |
| chr4 | 84457066 | | 84527027 | | NM_001256422 | | 1.48E-03 | | + | | *AGPAT9* | | 0.488703164199,0.164639013298 | | no | | 0 | |  |
| chr11 | 104813593 | | 104839325 | | NM_001225 | | 1.49E-03 | | - | | *CASP4* | | 0.398519041 | | yes | | 1 | |  |
| chr4 | 81106423 | | 81125482 | | NM_020226 | | 1.49E-03 | | + | | *PRDM8* | | 0.047453737 | | no | | 11 | |  |
| chr8 | 32579350 | | 32622558 | | NM_001159996 | | 1.50E-03 | | + | | *NRG1* | | 0.249573953724,0.197503297219 | | yes | | 4 | |  |
| chr19 | 55741146 | | 55770038 | | NM_014931 | | 1.50E-03 | | - | | *PPP6R1* | | 0.158500108678,0.429987130419,0.0592493778171 | | no | | 21 | |  |
| chr12 | 6666035 | | 6677498 | | NM_001033714 | | 1.51E-03 | | - | | *NOP2* | | 0.393765391532,0.295230166924 | | no | | 7 | |  |
| chr3 | 87276412 | | 87304698 | | NM_014043 | | 1.53E-03 | | + | | *CHMP2B* | | 0.317905853 | | no | | 5 | |  |
| chrX | 106045918 | | 106119377 | | NM_017752 | | 1.54E-03 | | + | | *TBC1D8B* | | 0.271987274 | | no | | 6 | |  |
| chr9 | 120466452 | | 120479769 | | NM_003266 | | 1.54E-03 | | + | | *TLR4* | | 0.572352625 | | yes | | 1 | |  |
| chr19 | 40476911 | | 40487671 | | NM_153001 | | 1.56E-03 | | + | | *PSMC4* | | 0.234570288094,0.249075304632 | | yes | | 3 | |  |
| chr7 | 66147077 | | 66276448 | | NM_001287060 | | 1.59E-03 | | + | | *RABGEF1* | | 0.304221264067,0.334205234556 | | no | | 8 | |  |
| chr10 | 22605311 | | 22609246 | | NM_012071 | | 1.62E-03 | | + | | *COMMD3* | | 0.334874313881,0.303006809539 | | no | | 4 | |  |
| chr8 | 97238903 | | 97247862 | | NM_006294 | | 1.62E-03 | | - | | *UQCRB* | | 0.345072755043,0.323679969433 | | no | | 2 | |  |
| chr2 | 190648810 | | 190742355 | | NM_001128143 | | 1.63E-03 | | + | | *PMS1* | | 0.495593126 | | no | | 6 | |  |
| chr2 | 27719705 | | 27746550 | | NM_001486 | | 1.63E-03 | | + | | *GCKR* | | 0.45566381 | | no | | 8 | |  |
| chr6 | 106959729 | | 107018324 | | NM_001624 | | 1.65E-03 | | + | | *AIM1* | | 0.076535545 | | no | | 0 | |  |
| chr1 | 109941652 | | 109969070 | | NM_002790 | | 1.66E-03 | | - | | *PSMA5* | | 0.357006960569,0.19908966059 | | yes | | 12 | |  |
| chr1 | 19923470 | | 19956315 | | NR_033757 | | 1.66E-03 | | + | | *MINOS1* | | 0.288228993 | | no | | 6 | |  |
| chr5 | 146770370 | | 146889619 | | NM_001197294 | | 1.67E-03 | | - | | *DPYSL3* | | 0.345072755 | | no | | 3 | |  |
| chr1 | 901876 | | 910484 | | NM_001160184 | | 1.67E-03 | | + | | *PLEKHN1* | | 0.381364373 | | no | | 18 | |  |
| chr16 | 699362 | | 717829 | | NM_145294 | | 1.67E-03 | | + | | *WDR90* | | 0.283087285 | | no | | 17 | |  |
| chr11 | 76927602 | | 76998463 | | NM_182833 | | 1.67E-03 | | - | | *GDPD4* | | 0.459323741 | | no | | 3 | |  |
| chr1 | 45805341 | | 45809650 | | NM_025077 | | 1.67E-03 | | + | | *TOE1* | | 0.41644536602,0.179783846944 | | no | | 9 | |  |
| chr1 | 32687970 | | 32697205 | | NM_003757 | | 1.68E-03 | | + | | *EIF3I* | | 0.369353906 | | no | | 5 | |  |
| chr20 | 3776385 | | 3786768 | | NM_021873 | | 1.68E-03 | | + | | *CDC25B* | | 0.495593125693,0.0720784622388 | | no | | 11 | |  |
| chr3 | 44803208 | | 44894748 | | NM_020242 | | 1.69E-03 | | + | | *KIF15* | | 0.292292577681,0.306664795272 | | no | | 7 | |  |
| chr1 | 32757707 | | 32799224 | | NM_004964 | | 1.70E-03 | | + | | *HDAC1* | | 0.068975769688,0.247090651392 | | yes | | 8 | |  |
| chr8 | 119935795 | | 119964383 | | NM_002546 | | 1.70E-03 | | - | | *TNFRSF11B* | | 0.229466093761,0.426560956345 | | no | | 2 | |  |
| chr4 | 83814604 | | 83822069 | | NR_034076 | | 1.70E-03 | | - | | *THAP9-AS1* | | 0.271443843 | | no | | 4 | |  |
| chr4 | 4269428 | | 4291748 | | NM_001145725 | | 1.73E-03 | | - | | *LYAR* | | 0.542265253 | | no | | 4 | |  |
| chr9 | 111934253 | | 112083244 | | NM_019114 | | 1.73E-03 | | - | | *EPB41L4B* | | 0.212247974 | | no | | 3 | |  |
| chr1 | 40627040 | | 40706593 | | NM_012421 | | 1.73E-03 | | + | | *RLF* | | 0.320459299925,0.358437848279 | | no | | 8 | |  |
| chr1 | 42312859 | | 42384496 | | NR_038260 | | 1.76E-03 | | - | | *HIVEP3* | | 0.072367353485,0.0211947875038 | | no | | 6 | |  |
| chr8 | 144694787 | | 144699732 | | NM_003313 | | 1.79E-03 | | - | | *TSTA3* | | 0.328243358191,0.181590704645 | | no | | 18 | |  |
| chr2 | 47129008 | | 47143007 | | NM_139279 | | 1.79E-03 | | - | | *MCFD2* | | 0.0130365282034,0.0572687602655,0.345763591159,0.226728956306 | | no | | 3 | |  |
| chr7 | 107564245 | | 107643804 | | NM_002291 | | 1.81E-03 | | - | | *LAMB1* | | 0.09671372 | | no | | 2 | |  |
| chr1 | 43629844 | | 43637986 | | NM_006824 | | 1.85E-03 | | - | | *EBNA1BP2* | | 0.36421898 | | no | | 5 | |  |
| chr5 | 179041178 | | 179050722 | | NM_005520 | | 1.86E-03 | | - | | *HNRNPH1* | | 0.213952768 | | no | | 0 | |  |
| chr15 | 41624891 | | 41673248 | | NM_016359 | | 1.86E-03 | | + | | *NUSAP1* | | 0.332871084 | | no | | 7 | |  |
| chr2 | 39208689 | | 39347604 | | NM_005633 | | 1.86E-03 | | - | | *SOS1* | | 0.024575629 | | yes | | 3 | |  |
| chr19 | 49949549 | | 49955115 | | NM_017916 | | 1.86E-03 | | - | | *PIH1D1* | | 0.389068444877,0.0490949058536 | | yes | | 15 | |  |
| chr3 | 120347014 | | 120401418 | | NM_000187 | | 1.86E-03 | | - | | *HGD* | | 0.0668034869468,0.563267855122 | | no | | 1 | |  |
| chr3 | 11267668 | | 11304939 | | NM_001098211 | | 1.87E-03 | | + | | *HRH1* | | 0.277481781556,0.307278738601 | | no | | 10 | |  |
| chr1 | 36771993 | | 36786948 | | NM_001162530 | | 1.87E-03 | | + | | *SH3D21* | | 0.395343607 | | no | | 14 | |  |
| chr4 | 17812435 | | 17846487 | | NM_022346 | | 1.88E-03 | | + | | *NCAPG* | | 0.348540794 | | no | | 5 | |  |
| chr5 | 95220801 | | 95297775 | | NM_012081 | | 1.88E-03 | | - | | *ELL2* | | 0.268205983 | | no | | 4 | |  |
| chr1 | 156638555 | | 156647189 | | NM_006617 | | 1.88E-03 | | - | | *NES* | | 0.182683524 | | yes | | 16 | |  |
| chr17 | 62120389 | | 62207502 | | NM_001433 | | 1.89E-03 | | - | | *ERN1* | | 0.239308922 | | yes | | 8 | |  |
| chr7 | 116164838 | | 116201239 | | NM_001753 | | 1.91E-03 | | + | | *CAV1* | | 0.380602407 | | yes | | 6 | |  |
| chr9 | 35736862 | | 35749225 | | NM_020944 | | 1.91E-03 | | - | | *GBA2* | | 0.190519638593,0.423162082318 | | no | | 10 | |  |
| chr12 | 30948614 | | 30955645 | | NR_040245 | | 1.94E-03 | | + | | *LINC00941* | | 0.0121551783299,0.0741251795061,0.270360236498,0.0324517794715 | | no | | 13 | |  |
| chr17 | 80901666 | | 81009686 | | NM_001009905 | | 1.94E-03 | | - | | *B3GNTL1* | | 0.361316851724,0.198294892543,0.0537183834459 | | no | | 14 | |  |
| chr2 | 234745346 | | 234763212 | | NM_001282963 | | 1.95E-03 | | - | | *HJURP* | | 0.230385796315,0.0887439519492 | | no | | 7 | |  |
| chrX | 119005733 | | 119010629 | | NM_004541 | | 1.95E-03 | | + | | *NDUFA1* | | 0.232701212062,0.323679969433 | | no | | 2 | |  |
| chr2 | 158114339 | | 158167913 | | NM_014568 | | 1.96E-03 | | + | | *GALNT5* | | 0.13212590305,0.170673995696 | | no | | 4 | |  |
| chr1 | 32372021 | | 32403988 | | NM_080391 | | 1.96E-03 | | - | | *PTP4A2* | | 0.171358058893,0.039163895099 | | no | | 8 | |  |
| chr1 | 52497768 | | 52521843 | | NR_046406 | | 1.98E-03 | | - | | *TXNDC12* | | 0.153815723 | | yes | | 5 | |  |
| chr2 | 58273776 | | 58387055 | | NM_001130483 | | 1.98E-03 | | + | | *VRK2* | | 0.151677306259,0.373065974367 | | no | | 5 | |  |
| chr14 | 20833825 | | 20881579 | | NM_007110 | | 1.99E-03 | | - | | *TEP1* | | 0.457490115 | | no | | 2 | |  |
| chr9 | 108006893 | | 108159628 | | NM_001286730 | | 2.00E-03 | | + | | *SLC44A1* | | 0.246596964 | | no | | 1 | |  |
| chr1 | 231154703 | | 231175995 | | NM_198552 | | 2.00E-03 | | - | | *FAM89A* | | 0.13917822123,0.388291085606 | | no | | 5 | |  |
| chr12 | 72003378 | | 72057749 | | NM_144982 | | 2.02E-03 | | - | | *ZFC3H1* | | 0.12568204672,0.313486180883,0.208045182357 | | no | | 4 | |  |
| chr1 | 32573643 | | 32642168 | | NM_012316 | | 2.04E-03 | | + | | *KPNA6* | | 0.42063071153,0.193205671895 | | no | | 8 | |  |
| chr9 | 140354403 | | 140445021 | | NM_001098537 | | 2.06E-03 | | - | | *PNPLA7* | | 0.25975926 | | no | | 22 | |  |
| chr19 | 40953690 | | 40971725 | | NM_000713 | | 2.06E-03 | | - | | *BLVRB* | | 0.0195261633977,0.163000827745 | | no | | 9 | |  |
| chr14 | 51462784 | | 51562422 | | NM_052978 | | 2.06E-03 | | - | | *TRIM9* | | 0.523090913 | | no | | 3 | |  |
| chr17 | 61904769 | | 61909387 | | NM_002805 | | 2.06E-03 | | + | | *PSMC5* | | 0.581583921 | | no | | 6 | |  |
| chr12 | 69201951 | | 69239324 | | NM_001145339 | | 2.07E-03 | | + | | *MDM2* | | 0.423162082 | | yes | | 4 | |  |
| chr1 | 6245079 | | 6259679 | | NM_000983 | | 2.09E-03 | | - | | *RPL22* | | 0.418114483 | | no | | 5 | |  |
| chr3 | 142315228 | | 142432505 | | NM_001145319 | | 2.09E-03 | | + | | *PLS1* | | 0.0959430972722,0.336216493707 | | no | | 9 | |  |
| chr1 | 28764660 | | 28826881 | | NM_023923 | | 2.09E-03 | | + | | *PHACTR4* | | 0.278037300453,0.330218738555 | | no | | 4 | |  |
| chr4 | 146054801 | | 146100832 | | NM_001102653 | | 2.10E-03 | | - | | *OTUD4* | | 0.382892885975,0.204742947763 | | no | | 2 | |  |
| chr19 | 35645844 | | 35660788 | | NM_001164605 | | 2.15E-03 | | + | | *FXYD5* | | 0.559898367 | | no | | 21 | |  |
| chr7 | 116312458 | | 116438440 | | NM_000245 | | 2.15E-03 | | + | | *MET* | | 0.594520548 | | no | | 5 | |  |
| chr4 | 184427234 | | 184433581 | | NM_001291959 | | 2.16E-03 | | + | | *ING2* | | 0.42485812 | | yes | | 8 | |  |
| chr17 | 79801033 | | 79818544 | | NM_000918 | | 2.16E-03 | | - | | *P4HB* | | 0.123687135817,0.336216493707,0.128477691079 | | no | | 16 | |  |
| chr1 | 28296854 | | 28415148 | | NM_001282562 | | 2.17E-03 | | - | | *EYA3* | | 0.425708687 | | no | | 3 | |  |
| chr2 | 220094609 | | 220101391 | | NM_001042410 | | 2.21E-03 | | + | | *ANKZF1* | | 0.439551671 | | no | | 15 | |  |
| chr11 | 66615996 | | 66725847 | | NM_000920 | | 2.21E-03 | | - | | *PC* | | 0.101875887 | | no | | 17 | |  |
| chr14 | 20937537 | | 20946165 | | NM_000270 | | 2.23E-03 | | + | | *PNP* | | 0.300592425 | | no | | 8 | |  |
| chr17 | 77071018 | | 77084685 | | NM_001042573 | | 2.23E-03 | | + | | *ENGASE* | | 0.452938013 | | no | | 13 | |  |
| chr8 | 74202873 | | 74205869 | | NM_000971 | | 2.23E-03 | | - | | *RPL7* | | 0.449328964117,0.0184258630639 | | no | | 2 | |  |
| chr3 | 195776154 | | 195808961 | | NM_003234 | | 2.24E-03 | | - | | *TFRC* | | 0.353454682 | | no | | 5 | |  |
| chr5 | 55147206 | | 55213165 | | NM_139017 | | 2.25E-03 | | + | | *IL31RA* | | 0.215240343171,0.349937749111 | | no | | 2 | |  |
| chr8 | 25285363 | | 25315984 | | NM_017634 | | 2.26E-03 | | - | | *KCTD9* | | 0.181227886 | | no | | 3 | |  |
| chr12 | 110288747 | | 110318293 | | NM_016433 | | 2.27E-03 | | - | | *GLTP* | | 0.220909977959,0.0695297889678,0.0157644164849 | | no | | 6 | |  |
| chr7 | 76940067 | | 77045717 | | NM_017439 | | 2.28E-03 | | - | | *GSAP* | | 0.352043687 | | no | | 6 | |  |
| chr1 | 26496387 | | 26497364 | | NM_015871 | | 2.29E-03 | | + | | *ZNF593* | | 0.326279795 | | no | | 14 | |  |
| chr12 | 21621843 | | 21654603 | | NM_032941 | | 2.31E-03 | | - | | *RECQL* | | 0.323033256422,0.29939245731 | | no | | 1 | |  |
| chr12 | 76419226 | | 76425556 | | NM_007350 | | 2.36E-03 | | - | | *PHLDA1* | | 0.0377790712622,0.186001600587,0.148080386595 | | yes | | 4 | |  |
| chr7 | 100849257 | | 100861011 | | NM_001084 | | 2.36E-03 | | - | | *PLOD3* | | 0.462087967584,0.163000827745 | | no | | 15 | |  |
| chr12 | 9436252 | | 9466684 | | NR_024374 | | 2.36E-03 | | + | | *LOC642846* | | 0.441313399 | | no | | 4 | |  |
| chr10 | 17686123 | | 17758821 | | NR_037774 | | 2.38E-03 | | + | | *STAM* | | 0.0844158583406,0.287078379846,0.0179529649395 | | no | | 0 | |  |
| chr7 | 86781676 | | 86825648 | | NM_001142326 | | 2.41E-03 | | + | | *DMTF1* | | 0.319819021816,0.0940432966592 | | no | | 2 | |  |
| chr9 | 139296373 | | 139305054 | | NM_001039707 | | 2.43E-03 | | - | | *SDCCAG3* | | 0.353454682 | | no | | 19 | |  |
| chr2 | 64319785 | | 64371605 | | NM_020651 | | 2.43E-03 | | - | | *PELI1* | | 0.341639223762,0.260279298379 | | no | | 4 | |  |
| chr20 | 2442280 | | 2451499 | | NM_198216 | | 2.45E-03 | | - | | *SNRPB* | | 0.263421465254,0.276374071328 | | no | | 8 | |  |
| chr1 | 109606997 | | 109618624 | | NM_005645 | | 2.45E-03 | | - | | *TAF13* | | 0.37832563 | | no | | 3 | |  |
| chr11 | 88053980 | | 88070941 | | NM_001114173 | | 2.47E-03 | | - | | *CTSC* | | 0.328900502 | | yes | | 4 | |  |
| chr20 | 3190133 | | 3204516 | | NM_181493 | | 2.52E-03 | | + | | *ITPA* | | 0.572352625 | | no | | 6 | |  |
| chr19 | 36103645 | | 36116251 | | NM_015302 | | 2.53E-03 | | + | | *HAUS5* | | 0.411477886 | | no | | 11 | |  |
| chr1 | 109822175 | | 109825790 | | NM_032636 | | 2.54E-03 | | - | | *PSRC1* | | 0.304221264 | | no | | 12 | |  |
| chr2 | 181845111 | | 181928154 | | NM_001278555 | | 2.54E-03 | | + | | *UBE2E3* | | 0.354871332 | | no | | 6 | |  |
| chr1 | 155232658 | | 155243320 | | NM_001294338 | | 2.55E-03 | | - | | *CLK2* | | 0.267670107 | | no | | 8 | |  |
| chr17 | 74261285 | | 74267379 | | NM_182565 | | 2.59E-03 | | + | | *UBALD2* | | 0.086121172 | | no | | 8 | |  |
| chr11 | 65190268 | | 65194003 | | NR_028272 | | 2.60E-03 | | + | | *NEAT1* | | 0.0134873914045,0.291708576721 | | no | | 8 | |  |
| chr18 | 77724581 | | 77730822 | | NM_001136180 | | 2.61E-03 | | + | | *HSBP1L1* | | 0.070088258 | | no | | 15 | |  |
| chr4 | 153547265 | | 153601317 | | NM_152680 | | 2.61E-03 | | - | | *TMEM154* | | 0.543350869074,0.0316189074594 | | no | | 6 | |  |
| chr1 | 150266261 | | 150281414 | | NM_031901 | | 2.62E-03 | | + | | *MRPS21* | | 0.336216493707,0.25872229826 | | no | | 5 | |  |
| chr12 | 109015679 | | 109027670 | | NM_003006 | | 2.62E-03 | | - | | *SELPLG* | | 0.42485812 | | no | | 11 | |  |
| chr1 | 63249776 | | 63330941 | | NM_032852 | | 2.64E-03 | | + | | *ATG4C* | | 0.452033042 | | no | | 2 | |  |
| chr1 | 223889294 | | 223963720 | | NM_001146068 | | 2.64E-03 | | + | | *CAPN2* | | 0.392193475894,0.148376843727,0.048801218362 | | no | | 4 | |  |
| chr4 | 147175132 | | 147443123 | | NM_001300842 | | 2.66E-03 | | - | | *SLC10A7* | | 0.298794270781,0.159453968047 | | no | | 5 | |  |
| chr1 | 24117645 | | 24122029 | | NM_007260 | | 2.66E-03 | | + | | *LYPLA2* | | 0.110140329886,0.027820002573 | | no | | 12 | |  |
| chr1 | 36805219 | | 36851528 | | NM_032017 | | 2.70E-03 | | - | | *STK40* | | 0.118599856781,0.254106959553,0.0245265273059 | | yes | | 15 | |  |
| chr1 | 117602948 | | 117645491 | | NM_003594 | | 2.78E-03 | | + | | *TTF2* | | 0.409016411 | | no | | 7 | |  |
| chr1 | 53692563 | | 53704282 | | NM_002370 | | 2.80E-03 | | - | | *MAGOH* | | 0.328243358 | | no | | 8 | |  |
| chr20 | 16347487 | | 16554079 | | NM_001199866 | | 2.81E-03 | | - | | *KIF16B* | | 0.27417189919,0.0771502854689,0.0185738611715 | | no | | 3 | |  |
| chr1 | 1682670 | | 1690081 | | NM_001198995 | | 2.81E-03 | | - | | *NADK* | | 0.537944438 | | no | | 19 | |  |
| chr1 | 27248212 | | 27273362 | | NM_006600 | | 2.86E-03 | | + | | *NUDC* | | 0.411477886 | | no | | 9 | |  |
| chr1 | 25568739 | | 25573985 | | NM_020317 | | 2.87E-03 | | - | | *RSRP1* | | 0.404946629 | | no | | 5 | |  |
| chr12 | 104324111 | | 104341708 | | NM_003299 | | 2.88E-03 | | + | | *HSP90B1* | | 0.103933916508,0.233167080199 | | yes | | 12 | |  |
| chr17 | 38544772 | | 38574202 | | NM_001067 | | 2.90E-03 | | - | | *TOP2A* | | 0.423162082 | | no | | 3 | |  |
| chr1 | 43824625 | | 43828873 | | NM_001255 | | 2.90E-03 | | + | | *CDC20* | | 0.362040209 | | no | | 8 | |  |
| chr7 | 77313167 | | 77326662 | | NR_038361 | | 2.90E-03 | | - | | *APTR* | | 0.046887695 | | no | | 0 | |  |
| chr1 | 67873492 | | 67896123 | | NM_015640 | | 2.92E-03 | | - | | *SERBP1* | | 0.253599254 | | yes | | 10 | |  |
| chr11 | 66452719 | | 66488870 | | NM_006946 | | 2.97E-03 | | - | | *SPTBN2* | | 0.0352954930088,0.252082213659 | | no | | 4 | |  |
| chr16 | 30996518 | | 31000473 | | NM_001142778 | | 2.99E-03 | | + | | *HSD3B7* | | 0.099659092 | | no | | 23 | |  |
| chr2 | 201170984 | | 201346986 | | NM_001100422 | | 2.99E-03 | | + | | *SPATS2L* | | 0.438673447 | | no | | 7 | |  |
| chr12 | 7085346 | | 7125842 | | NM_005768 | | 2.99E-03 | | - | | *LPCAT3* | | 0.344383299 | | no | | 9 | |  |
| chr1 | 879582 | | 894679 | | NM_015658 | | 3.01E-03 | | - | | *NOC2L* | | 0.341639224 | | yes | | 17 | |  |
| chr11 | 77033059 | | 77185108 | | NM_001128620 | | 3.02E-03 | | - | | *PAK1* | | 0.0916296838775,0.154432217592 | | yes | | 6 | |  |
| chr17 | 7835441 | | 7853237 | | NM_001037144 | | 3.04E-03 | | + | | *CNTROB* | | 0.319819022 | | no | | 14 | |  |
| chr12 | 6643570 | | 6647541 | | NM_002046 | | 3.04E-03 | | + | | *GAPDH* | | 0.0788663997907,0.389847360423,0.048801218362 | | no | | 17 | |  |
| chr7 | 30323922 | | 30407308 | | NM_147128 | | 3.05E-03 | | + | | *ZNRF2* | | 0.382892886 | | no | | 12 | |  |
| chr2 | 75719443 | | 75788092 | | NM_001135032 | | 3.06E-03 | | - | | *EVA1A* | | 0.396928149 | | no | | 4 | |  |
| chr7 | 42956461 | | 42971805 | | NM_002787 | | 3.11E-03 | | - | | *PSMA2* | | 0.329558961 | | yes | | 8 | |  |
| chr1 | 955502 | | 991499 | | NM_198576 | | 3.11E-03 | | + | | *AGRN* | | 0.034252354 | | yes | | 19 | |  |
| chrX | 100645877 | | 100651142 | | NM_021029 | | 3.13E-03 | | + | | *RPL36A* | | 0.239308922 | | no | | 4 | |  |
| chr4 | 39460643 | | 39465434 | | NM_001278592 | | 3.13E-03 | | + | | *LIAS* | | 0.294051604952,0.252082213659 | | no | | 7 | |  |
| chr22 | 50656117 | | 50683400 | | NM_020461 | | 3.15E-03 | | - | | *TUBGCP6* | | 0.42998713 | | no | | 17 | |  |
| chr5 | 90606730 | | 90610219 | | NR_103549 | | 3.16E-03 | | - | | *LUCAT1* | | 0.0303791123645,0.304221264067,0.12518032265 | | no | | 6 | |  |
| chr1 | 209788217 | | 209824679 | | NM_001017402 | | 3.16E-03 | | - | | *LAMB3* | | 0.0297775656359,0.364948146454,0.0306231199925 | | no | | 11 | |  |
| chr11 | 64894750 | | 64902003 | | NM_032431 | | 3.16E-03 | | - | | *SYVN1* | | 0.431710523 | | no | | 10 | |  |
| chr12 | 113659242 | | 113736389 | | NM_001143819 | | 3.17E-03 | | + | | *TPCN1* | | 0.191283243339,0.0849238760196 | | no | | 7 | |  |
| chr4 | 175411327 | | 175444049 | | NM_000860 | | 3.18E-03 | | - | | *HPGD* | | 0.542265253 | | no | | 1 | |  |
| chr20 | 5095598 | | 5100647 | | NM_182649 | | 3.19E-03 | | - | | *PCNA* | | 0.119791805158,0.390627835359 | | no | | 11 | |  |
| chr2 | 182756442 | | 182795464 | | NM_006751 | | 3.21E-03 | | + | | *SSFA2* | | 0.334874314 | | no | | 5 | |  |
| chr16 | 15737123 | | 15820208 | | NM_001143979 | | 3.23E-03 | | + | | *NDE1* | | 0.419790290808,0.066536806715,0.0177033746391 | | no | | 3 | |  |
| chr7 | 1570367 | | 1582679 | | NM_002360 | | 3.23E-03 | | + | | *MAFK* | | 0.038465252 | | no | | 18 | |  |
| chr1 | 46153846 | | 46160108 | | NM_016486 | | 3.25E-03 | | + | | *TMEM69* | | 0.045959256649,0.235039898123 | | no | | 5 | |  |
| chr17 | 74380689 | | 74383941 | | NM_182965 | | 3.28E-03 | | + | | *SPHK1* | | 0.318542301 | | yes | | 18 | |  |
| chrX | 118967988 | | 118986991 | | NM_023010 | | 3.29E-03 | | - | | *UPF3B* | | 0.414782912 | | no | | 6 | |  |
| chr1 | 1447522 | | 1470067 | | NM_001170535 | | 3.31E-03 | | + | | *ATAD3A* | | 0.273624103 | | yes | | 16 | |  |
| chr1 | 28832454 | | 28837404 | | NR_036473 | | 3.32E-03 | | + | | *SNHG3* | | 0.280831622 | | no | | 5 | |  |
| chr12 | 110890290 | | 110906526 | | NM_001164372 | | 3.32E-03 | | - | | *GPN3* | | 0.452938013 | | no | | 5 | |  |
| chr2 | 239969863 | | 240322643 | | NM_006037 | | 3.33E-03 | | - | | *HDAC4* | | 0.187120965 | | yes | | 5 | |  |
| chr8 | 67474409 | | 67525484 | | NM_001144755 | | 3.34E-03 | | - | | *MYBL1* | | 0.234101616 | | no | | 5 | |  |
| chr1 | 47715810 | | 47779819 | | NM_001048166 | | 3.34E-03 | | - | | *STIL* | | 0.357006961 | | no | | 2 | |  |
| chr19 | 47759730 | | 47775210 | | NM_015603 | | 3.36E-03 | | + | | *CCDC9* | | 0.349937749111,0.0416689096582 | | no | | 12 | |  |
| chr17 | 8128138 | | 8151413 | | NM_025099 | | 3.38E-03 | | - | | *CTC1* | | 0.343695220928,0.136695425446,0.030439931388 | | no | | 6 | |  |
| chr12 | 89741601 | | 89746636 | | NM_001946 | | 3.39E-03 | | - | | *DUSP6* | | 0.0475487394658,0.229466093761,0.0112431075535 | | yes | | 1 | |  |
| chr17 | 71279762 | | 71308143 | | NM_012121 | | 3.41E-03 | | - | | *CDC42EP4* | | 0.285361066658,0.223576867027 | | no | | 11 | |  |
| chr16 | 29827527 | | 29833816 | | NM_024516 | | 3.42E-03 | | + | | *PAGR1* | | 0.475209272 | | no | | 9 | |  |
| chr8 | 23048969 | | 23082680 | | NM_003844 | | 3.44E-03 | | - | | *TNFRSF10A* | | 0.486752256 | | no | | 6 | |  |
| chrY | 21867300 | | 21906825 | | NM_001146706 | | 3.48E-03 | | - | | *KDM5D* | | 0.345763591 | | no | | 5 | |  |
| chr14 | 31028328 | | 31089046 | | NM_017769 | | 3.51E-03 | | + | | *G2E3* | | 0.393765392 | | no | | 3 | |  |
| chr2 | 33359663 | | 33624575 | | NM_001166266 | | 3.52E-03 | | + | | *LTBP1* | | 0.379083038 | | no | | 7 | |  |
| chr1 | 28879528 | | 28905057 | | NM_017846 | | 3.53E-03 | | + | | *TRNAU1AP* | | 0.275270783 | | no | | 3 | |  |
| chrX | 114238537 | | 114252207 | | NM_000640 | | 3.54E-03 | | - | | *IL13RA2* | | 0.431710523429,0.0266757597627 | | no | | 4 | |  |
| chr8 | 87354993 | | 87480178 | | NM_007013 | | 3.54E-03 | | + | | *WWP1* | | 0.334874313881,0.0256810991316 | | no | | 8 | |  |
| chr3 | 50606908 | | 50622421 | | NM_016173 | | 3.58E-03 | | + | | *HEMK1* | | 0.348540794 | | no | | 13 | |  |
| chr11 | 120081746 | | 120100650 | | NM_178507 | | 3.58E-03 | | + | | *OAF* | | 0.173773943 | | no | | 12 | |  |
| chr12 | 75891418 | | 75905418 | | NM_007043 | | 3.58E-03 | | - | | *KRR1* | | 0.306664795 | | no | | 3 | |  |
| chr4 | 152041432 | | 152147660 | | NM_001128923 | | 3.60E-03 | | - | | *SH3D19* | | 0.315372752022,0.0593679951505 | | no | | 3 | |  |
| chr19 | 17862285 | | 17899377 | | NM_001161359 | | 3.61E-03 | | + | | *FCHO1* | | 0.40011631 | | no | | 5 | |  |
| chr7 | 66452689 | | 66460588 | | NM_016038 | | 3.66E-03 | | - | | *SBDS* | | 0.029363584 | | no | | 2 | |  |
| chr22 | 50354142 | | 50357720 | | NM_001001852 | | 3.66E-03 | | + | | *PIM3* | | 0.273624103 | | yes | | 18 | |  |
| chr8 | 62413114 | | 62602408 | | NM_001164750 | | 3.67E-03 | | - | | *ASPH* | | 0.0295994351679,0.405757333019 | | no | | 4 | |  |
| chr1 | 110091185 | | 110138454 | | NM_006496 | | 3.68E-03 | | + | | *GNAI3* | | 0.020040501 | | no | | 7 | |  |
| chr7 | 28725597 | | 28865511 | | NM_001011666 | | 3.70E-03 | | + | | *CREB5* | | 0.240749092 | | no | | 4 | |  |
| chr3 | 50126340 | | 50156397 | | NM_005778 | | 3.71E-03 | | + | | *RBM5* | | 0.409835262 | | no | | 6 | |  |
| chr1 | 12040237 | | 12073572 | | NM_014874 | | 3.73E-03 | | + | | *MFN2* | | 0.197108685368,0.238830782698 | | no | | 6 | |  |
| chr19 | 50162825 | | 50169132 | | NM_001197128 | | 3.75E-03 | | - | | *IRF3* | | 0.185629969 | | yes | | 18 | |  |
| chr17 | 73906617 | | 73937119 | | NM_001080542 | | 3.76E-03 | | - | | *FBF1* | | 0.415613308 | | no | | 0 | |  |
| chr9 | 127997126 | | 128003666 | | NM_005347 | | 3.80E-03 | | - | | *HSPA5* | | 0.228093419 | | yes | | 6 | |  |
| chr1 | 38000049 | | 38019945 | | NM_024700 | | 3.85E-03 | | - | | *SNIP1* | | 0.36059494 | | no | | 6 | |  |
| chr1 | 75198861 | | 75232360 | | NR_027962 | | 3.90E-03 | | + | | *TYW3* | | 0.0121551783299,0.359155441329 | | no | | 10 | |  |
| chr1 | 24290836 | | 24306953 | | NM_001191005 | | 3.96E-03 | | - | | *SRSF10* | | 0.281393847 | | no | | 2 | |  |
| chr7 | 77428108 | | 77586821 | | NM_001127358 | | 3.98E-03 | | + | | *PHTF2* | | 0.192049909 | | no | | 5 | |  |
| chr20 | 388708 | | 411610 | | NM_006462 | | 3.99E-03 | | + | | *RBCK1* | | 0.0910815518148,0.09273586389 | | yes | | 9 | |  |
| chr17 | 76108998 | | 76124861 | | NM_001127198 | | 4.02E-03 | | - | | *TMC6* | | 0.082249333 | | no | | 15 | |  |
| chr1 | 28218035 | | 28241308 | | NM_002946 | | 4.02E-03 | | - | | *RPA2* | | 0.374561227 | | no | | 4 | |  |
| chr1 | 43829067 | | 43833409 | | NM_001256399 | | 4.03E-03 | | - | | *ELOVL1* | | 0.27362410337,0.116251422271 | | no | | 9 | |  |
| chr1 | 101361633 | | 101447311 | | NM_133496 | | 4.03E-03 | | + | | *SLC30A7* | | 0.0837432255922,0.303006809539 | | no | | 8 | |  |
| chr18 | 20513294 | | 20606449 | | NM_002894 | | 4.04E-03 | | + | | *RBBP8* | | 0.379841963 | | no | | 2 | |  |
| chr1 | 93307716 | | 93427079 | | NM_001006605 | | 4.07E-03 | | - | | *FAM69A* | | 0.0162445144419,0.0878609348735,0.246596963942 | | no | | 2 | |  |
| chr11 | 129939715 | | 130014706 | | NM_001142277 | | 4.08E-03 | | + | | *APLP2* | | 0.159773195 | | no | | 18 | |  |
| chr3 | 9989087 | | 9996471 | | NR_046734 | | 4.09E-03 | | + | | *PRRT3-AS1* | | 0.0619145543569,0.210978298818 | | no | | 14 | |  |
| chr12 | 88442789 | | 88535993 | | NM_025114 | | 4.11E-03 | | - | | *CEP290* | | 0.372320588 | | no | | 2 | |  |
| chr1 | 9599527 | | 9642831 | | NM_032315 | | 4.12E-03 | | + | | *SLC25A33* | | 0.211400678 | | no | | 6 | |  |
| chr12 | 108955238 | | 108963160 | | NM_014301 | | 4.12E-03 | | + | | *ISCU* | | 0.216535667 | | no | | 8 | |  |
| chr1 | 948846 | | 949919 | | NM_005101 | | 4.13E-03 | | + | | *ISG15* | | 0.289963565529,0.0597252738858 | | no | | 15 | |  |
| chr11 | 66099541 | | 66104000 | | NM_004292 | | 4.13E-03 | | - | | *RIN1* | | 0.297006873 | | no | | 20 | |  |
| chr2 | 70142172 | | 70170076 | | NM_001202514 | | 4.13E-03 | | + | | *MXD1* | | 0.164310064 | | no | | 11 | |  |
| chr4 | 41258897 | | 41270446 | | NM_004181 | | 4.15E-03 | | + | | *UCHL1* | | 0.266068894994,0.169653017719 | | no | | 7 | |  |
| chr19 | 50359721 | | 50362546 | | NR_110730 | | 4.15E-03 | | - | | *PTOV1-AS2* | | 0.222684346 | | no | | 15 | |  |
| chr20 | 60962120 | | 60963576 | | NM_001024 | | 4.16E-03 | | + | | *RPS21* | | 0.362765014 | | no | | 13 | |  |
| chr1 | 156495196 | | 156542396 | | NM_178229 | | 4.17E-03 | | - | | *IQGAP3* | | 0.0244286171475,0.235039898123 | | no | | 9 | |  |
| chr7 | 50657759 | | 50861159 | | NM_001001555 | | 4.18E-03 | | - | | *GRB10* | | 0.258205371 | | no | | 4 | |  |
| chr2 | 48541794 | | 48606434 | | NM_002158 | | 4.19E-03 | | + | | *FOXN2* | | 0.199887614 | | no | | 6 | |  |
| chr1 | 46654352 | | 46664121 | | NM_017739 | | 4.20E-03 | | - | | *POMGNT1* | | 0.349238573 | | no | | 12 | |  |
| chr5 | 162887516 | | 162918953 | | NM_001142557 | | 4.20E-03 | | + | | *HMMR* | | 0.319180023 | | no | | 3 | |  |
| chrX | 99929488 | | 99987135 | | NM_001129896 | | 4.26E-03 | | - | | *SYTL4* | | 0.332871084 | | no | | 4 | |  |
| chr1 | 38422651 | | 38455761 | | NM_006802 | | 4.28E-03 | | - | | *SF3A3* | | 0.225823852 | | no | | 4 | |  |
| chr1 | 94027342 | | 94079702 | | NM_001261410 | | 4.29E-03 | | - | | *BCAR3* | | 0.036443214 | | no | | 4 | |  |
| chr14 | 23776068 | | 23780968 | | NM_001199839 | | 4.30E-03 | | + | | *BCL2L2* | | 0.309127953 | | no | | 7 | |  |
| chr17 | 80193649 | | 80197375 | | NM_001206952 | | 4.31E-03 | | + | | *SLC16A3* | | 0.227182868 | | no | | 23 | |  |
| chr7 | 77166772 | | 77269388 | | NM_002835 | | 4.33E-03 | | + | | *PTPN12* | | 0.102284207 | | no | | 7 | |  |
| chr19 | 36208920 | | 36229781 | | NM_014727 | | 4.34E-03 | | + | | *KMT2B* | | 0.0464211548574,0.328243358191 | | no | | 16 | |  |
| chr20 | 47894714 | | 47905795 | | NR_036658 | | 4.35E-03 | | + | | *ZFAS1* | | 0.434308573 | | no | | 15 | |  |
| chr3 | 57557089 | | 57583215 | | NM_001660 | | 4.35E-03 | | - | | *ARF4* | | 0.163654136803,0.143130282079 | | no | | 4 | |  |
| chr3 | 185361526 | | 185538919 | | NM_001291873 | | 4.36E-03 | | - | | *IGF2BP2* | | 0.409835262 | | no | | 7 | |  |
| chr3 | 122103022 | | 122128961 | | NM_014367 | | 4.38E-03 | | + | | *FAM162A* | | 0.178708374 | | no | | 7 | |  |
| chr2 | 241526132 | | 241538526 | | NM_023083 | | 4.40E-03 | | + | | *CAPN10* | | 0.387515279 | | no | | 10 | |  |
| chr7 | 44646120 | | 44748669 | | NM_001165036 | | 4.40E-03 | | + | | *OGDH* | | 0.158500109 | | no | | 4 | |  |
| chr11 | 69061604 | | 69064754 | | NM_138768 | | 4.42E-03 | | + | | *MYEOV* | | 0.075925704 | | no | | 11 | |  |
| chr3 | 142720371 | | 142779567 | | NM_001080415 | | 4.42E-03 | | + | | *U2SURP* | | 0.021025906 | | no | | 4 | |  |
| chr7 | 73245192 | | 73247023 | | NM_001305 | | 4.43E-03 | | + | | *CLDN4* | | 0.0165726754018,0.297006872713,0.0866394521154 | | no | | 10 | |  |
| chr7 | 100171633 | | 100183811 | | NM_001289934 | | 4.44E-03 | | - | | *LRCH4* | | 0.413127094 | | no | | 22 | |  |
| chr1 | 15479027 | | 15546974 | | NM_001136217 | | 4.51E-03 | | + | | *TMEM51* | | 0.292877748 | | no | | 18 | |  |
| chr16 | 28962317 | | 28977767 | | NM_032815 | | 4.63E-03 | | + | | *NFATC2IP* | | 0.157867375 | | no | | 7 | |  |
| chr1 | 89445138 | | 89458643 | | NM_019610 | | 4.67E-03 | | - | | *RBMXL1* | | 0.294640297 | | no | | 3 | |  |
| chr1 | 86115105 | | 86174116 | | NM_001170670 | | 4.67E-03 | | - | | *ZNHIT6* | | 0.345763591 | | no | | 2 | |  |
| chr7 | 95034173 | | 95064384 | | NM_000305 | | 4.68E-03 | | - | | *PON2* | | 0.25410696 | | no | | 3 | |  |
| chr2 | 102013822 | | 102091165 | | NM_001145664 | | 4.71E-03 | | - | | *RFX8* | | 0.190901059 | | no | | 7 | |  |
| chr11 | 64073698 | | 64084212 | | NM_001282450 | | 4.73E-03 | | + | | *ESRRA* | | 0.361316852 | | no | | 19 | |  |
| chr4 | 147096834 | | 147111213 | | NM_007080 | | 4.73E-03 | | + | | *LSM6* | | 0.403330078 | | no | | 4 | |  |
| chr1 | 70610484 | | 70671361 | | NM_017768 | | 4.76E-03 | | - | | *LRRC40* | | 0.410655753 | | no | | 2 | |  |
| chr15 | 72491369 | | 72523727 | | NM_182471 | | 4.77E-03 | | - | | *PKM* | | 0.323033256422,0.0414196447428 | | no | | 12 | |  |
| chr1 | 19400999 | | 19536746 | | NM_020765 | | 4.81E-03 | | - | | *UBR4* | | 0.168638147269,0.210136071201 | | no | | 14 | |  |
| chr17 | 8043787 | | 8055753 | | NM_002616 | | 4.83E-03 | | - | | *PER1* | | 0.171701117956,0.100459562062 | | no | | 8 | |  |
| chr5 | 148521571 | | 148640001 | | NM_001301018 | | 4.84E-03 | | + | | *ABLIM3* | | 0.296413453 | | no | | 9 | |  |
| chr4 | 72053002 | | 72437804 | | NM_001134742 | | 4.86E-03 | | + | | *SLC4A4* | | 0.385968314 | | no | | 12 | |  |
| chr12 | 71833549 | | 71980088 | | NM_001277226 | | 4.88E-03 | | + | | *LGR5* | | 0.312234741 | | no | | 10 | |  |
| chr16 | 8715526 | | 8740079 | | NM_024109 | | 4.89E-03 | | + | | *METTL22* | | 0.374561227 | | no | | 11 | |  |
| chr2 | 169929084 | | 169952677 | | NM_001142270 | | 4.90E-03 | | + | | *DHRS9* | | 0.0604462946447,0.0287246396542 | | no | | 0 | |  |
| chr4 | 74606222 | | 74609433 | | NM_000584 | | 4.91E-03 | | + | | *CXCL8* | | 0.213952768 | | no | | 1 | |  |
| chr17 | 40962149 | | 40976310 | | NM_003766 | | 4.95E-03 | | - | | *BECN1* | | 0.390627835 | | no | | 4 | |  |
| chr2 | 44502596 | | 44547962 | | NM_000341 | | 4.95E-03 | | + | | *SLC3A1* | | 0.336216494 | | no | | 1 | |  |
| chr8 | 99956630 | | 99964332 | | NM_001142462 | | 4.96E-03 | | + | | *OSR2* | | 0.210136071 | | no | | 10 | |  |
| chr1 | 93811477 | | 93828148 | | NM_001938 | | 4.99E-03 | | + | | *DR1* | | 0.203518168 | | no | | 10 | |  |
| chr1 | 68167148 | | 68299155 | | NM_018841 | | 4.99E-03 | | - | | *GNG12* | | 0.032193201 | | no | | 3 | |  |
| chr3 | 42824399 | | 42845934 | | NM_001099668 | | 5.03E-03 | | - | | *HIGD1A* | | 0.371576691 | | no | | 3 | |  |
| chr2 | 139259349 | | 139330805 | | NM_001001664 | | 5.04E-03 | | + | | *SPOPL* | | 0.306052079 | | no | | 7 | |  |
| chr1 | 8071778 | | 8086393 | | NM_018948 | | 5.05E-03 | | - | | *ERRFI1* | | 0.0955600914055,0.0239928358367 | | no | | 9 | |  |
| chr3 | 49758908 | | 49761407 | | NM_013334 | | 5.07E-03 | | - | | *GMPPB* | | 0.37832563 | | no | | 12 | |  |
| chr1 | 20978259 | | 20988037 | | NM_005216 | | 5.08E-03 | | - | | *DDOST* | | 0.309746828 | | no | | 7 | |  |
| chr1 | 38158072 | | 38175391 | | NM_018101 | | 5.12E-03 | | + | | *CDCA8* | | 0.352043687 | | no | | 10 | |  |
| chr15 | 74833547 | | 74890472 | | NM_006465 | | 5.13E-03 | | + | | *ARID3B* | | 0.228093419 | | no | | 9 | |  |
| chr11 | 64692142 | | 64701950 | | NM_006244 | | 5.19E-03 | | + | | *PPP2R5B* | | 0.365678773 | | no | | 14 | |  |
| chr7 | 99055783 | | 99063824 | | NM_004889 | | 5.23E-03 | | - | | *ATP5J2* | | 0.338917013 | | no | | 0 | |  |
| chr9 | 127020529 | | 127114719 | | NM_001166168 | | 5.24E-03 | | + | | *NEK6* | | 0.017283552 | | yes | | 15 | |  |
| chr19 | 1248551 | | 1259142 | | NM_177401 | | 5.24E-03 | | + | | *MIDN* | | 0.357006961 | | no | | 20 | |  |
| chr8 | 141541263 | | 141645646 | | NM_001164623 | | 5.29E-03 | | - | | *AGO2* | | 0.089099639 | | no | | 3 | |  |
| chr9 | 116029289 | | 116037869 | | NM_139286 | | 5.31E-03 | | - | | *CDC26* | | 0.338917013 | | no | | 1 | |  |
| chr4 | 153332311 | | 153456393 | | NM_001257069 | | 5.33E-03 | | - | | *FBXW7* | | 0.189759082166,0.0383883980176 | | no | | 2 | |  |
| chr2 | 47387220 | | 47403740 | | NM_001743 | | 5.33E-03 | | - | | *CALM2* | | 0.172044864 | | no | | 1 | |  |
| chr17 | 40761357 | | 40767256 | | NM_001070 | | 5.44E-03 | | + | | *TUBG1* | | 0.198294893 | | no | | 9 | |  |
| chr17 | 1614797 | | 1619566 | | NR_028502 | | 5.48E-03 | | - | | *MIR22HG* | | 0.0625368059732,0.174121839117,0.0350142555146 | | no | | 18 | |  |
| chr9 | 113006091 | | 113018920 | | NM_003329 | | 5.48E-03 | | - | | *TXN* | | 0.0341839172967,0.0611760197745 | | no | | 3 | |  |
| chr15 | 52873517 | | 52970831 | | NM_019600 | | 5.52E-03 | | - | | *FAM214A* | | 0.318542301 | | no | | 2 | |  |
| chr1 | 113243748 | | 113249678 | | NM_001042679 | | 5.52E-03 | | - | | *RHOC* | | 0.0184258630639,0.0573834123999 | | no | | 17 | |  |
| chr12 | 26272958 | | 26278003 | | NM_030762 | | 5.54E-03 | | - | | *BHLHE41* | | 0.235510448313,0.0382351511236 | | no | | 2 | |  |
| chr20 | 61427804 | | 61431945 | | NM_018270 | | 5.58E-03 | | + | | *MRGBP* | | 0.0187230480126,0.0497870683679 | | no | | 15 | |  |
| chr1 | 35899090 | | 36023037 | | NM_024874 | | 5.66E-03 | | - | | *KIAA0319L* | | 0.17728441 | | no | | 9 | |  |
| chr4 | 159593252 | | 159629865 | | NM_004453 | | 5.70E-03 | | + | | *ETFDH* | | 0.332871084 | | no | | 8 | |  |
| chr3 | 196728611 | | 196756687 | | NM_005929 | | 5.71E-03 | | - | | *MFI2* | | 0.042681061 | | no | | 0 | |  |
| chr11 | 128328655 | | 128392205 | | NM_005238 | | 5.74E-03 | | - | | *ETS1* | | 0.118599857 | | no | | 4 | |  |
| chr7 | 11013498 | | 11209250 | | NR_033436 | | 5.82E-03 | | + | | *PHF14* | | 0.274720792 | | no | | 7 | |  |
| chr2 | 208394615 | | 208470284 | | NM_134442 | | 5.84E-03 | | + | | *CREB1* | | 0.284790915 | | no | | 9 | |  |
| chr1 | 24742244 | | 24799473 | | NM_020448 | | 5.87E-03 | | + | | *NIPAL3* | | 0.023190791 | | no | | 7 | |  |
| chr4 | 114821439 | | 114900878 | | NM_024590 | | 5.92E-03 | | - | | *ARSJ* | | 0.265006745 | | no | | 2 | |  |
| chr2 | 217363519 | | 217366188 | | NM_000998 | | 5.93E-03 | | + | | *RPL37A* | | 0.267670107 | | no | | 7 | |  |
| chr8 | 118811601 | | 119124058 | | NM_000127 | | 5.94E-03 | | - | | *EXT1* | | 0.274171899 | | no | | 2 | |  |
| chr12 | 120427647 | | 120532299 | | NM_207311 | | 5.96E-03 | | + | | *CCDC64* | | 0.275270783 | | no | | 0 | |  |
| chr2 | 143635194 | | 143799885 | | NM_001199241 | | 5.97E-03 | | + | | *KYNU* | | 0.138069237311,0.0393995848317 | | no | | 3 | |  |
| chr1 | 150254942 | | 150259504 | | NM_001300838 | | 6.03E-03 | | + | | *CIART* | | 0.162025751 | | no | | 10 | |  |
| chr2 | 74648884 | | 74652882 | | NM_032118 | | 6.06E-03 | | + | | *WDR54* | | 0.292292578 | | no | | 7 | |  |
| chr4 | 119643977 | | 119757326 | | NM_014822 | | 6.08E-03 | | - | | *SEC24D* | | 0.103933917 | | no | | 1 | |  |
| chr7 | 100805789 | | 100808852 | | NM_003378 | | 6.10E-03 | | - | | *VGF* | | 0.0111312367641,0.0250219960237 | | no | | 10 | |  |
| chr16 | 577855 | | 604636 | | NM_005632 | | 6.28E-03 | | + | | *CAPN15* | | 0.158183425 | | no | | 13 | |  |
| chr9 | 123151146 | | 123342448 | | NR_073556 | | 6.29E-03 | | - | | *CDK5RAP2* | | 0.216535667 | | no | | 6 | |  |
| chr9 | 130965633 | | 131017527 | | NM_001288739 | | 6.34E-03 | | + | | *DNM1* | | 0.125682047 | | no | | 11 | |  |
| chr1 | 23631180 | | 23670857 | | NM_001102397 | | 6.37E-03 | | - | | *HNRNPR* | | 0.184519524 | | no | | 1 | |  |
| chr1 | 1167628 | | 1170420 | | NM_080605 | | 6.42E-03 | | + | | *B3GALT6* | | 0.262895149 | | no | | 7 | |  |
| chr17 | 45195062 | | 45266678 | | NM_001293091 | | 6.47E-03 | | - | | *CDC27* | | 0.231309185 | | no | | 0 | |  |
| chr20 | 17550598 | | 17588652 | | NM_006870 | | 6.62E-03 | | + | | *DSTN* | | 0.0227770916572,0.116019151774 | | no | | 13 | |  |
| chr20 | 57226308 | | 57254582 | | NM_001204868 | | 6.67E-03 | | + | | *STX16* | | 0.07965902 | | no | | 11 | |  |
| chr1 | 9352940 | | 9429590 | | NM_025106 | | 6.69E-03 | | + | | *SPSB1* | | 0.037179416 | | no | | 15 | |  |
| chr8 | 82192717 | | 82197012 | | NM_001444 | | 6.75E-03 | | + | | *FABP5* | | 0.225823852 | | no | | 13 | |  |
| chr19 | 8429010 | | 8439259 | | NM_139314 | | 6.82E-03 | | + | | *ANGPTL4* | | 0.0212797364384,0.114406200652 | | no | | 12 | |  |
| chr6 | 3269206 | | 3456793 | | NM_015482 | | 6.83E-03 | | - | | *SLC22A23* | | 0.117419768 | | no | | 10 | |  |
| chr17 | 74466974 | | 74497509 | | NM_024599 | | 6.86E-03 | | - | | *RHBDF2* | | 0.031304294 | | no | | 19 | |  |
| chr4 | 169277885 | | 169401665 | | NM_001012967 | | 6.88E-03 | | - | | *DDX60L* | | 0.180504423 | | no | | 2 | |  |
| chr3 | 141205888 | | 141334205 | | NM_001303245 | | 6.97E-03 | | + | | *RASA2* | | 0.069529789 | | no | | 9 | |  |
| chr12 | 27485786 | | 27578746 | | NM_001248005 | | 7.08E-03 | | + | | *ARNTL2* | | 0.101266462 | | no | | 5 | |  |
| chr1 | 155146262 | | 155157447 | | NM_001256599 | | 7.74E-03 | | + | | *TRIM46* | | 0.140296113 | | no | | 14 | |  |
| chr16 | 1988233 | | 1993294 | | NM_016332 | | 8.33E-03 | | - | | *MSRB1* | | 0.029658693 | | no | | 11 | |  |
| chr2 | 27532359 | | 27545969 | | NM_002437 | | 8.35E-03 | | - | | *MPV17* | | 0.03062312 | | no | | 14 | |  |
| chr1 | 78470635 | | 78482995 | | NM_007034 | | 8.44E-03 | | + | | *DNAJB4* | | 0.014094086 | | no | | 1 | |  |
| chr5 | 74017028 | | 74063196 | | NM_032380 | | 8.60E-03 | | - | | *GFM2* | | 0.024185548 | | no | | 5 | |  |
| chr8 | 122651585 | | 122657564 | | NR_002835 | | 8.64E-03 | | + | | *HAS2-AS1* | | 0.032193201 | | no | | 5 | |  |

* From combined analysis of different peaks of H3K27ac and differentially expressed genes.
