# Supplementary material for: A novel role of Krüppel-like factor 8 as an apoptosis repressor in hepatocellular carcinoma
Source: Cancer Cell Int. 2020 Aug 28;20:422. doi: 10.1186/s12935-020-01513-3 (PMC7456055; doi:10.1186/s12935-020-01513-3)
Supplement: Supplementary file 2 — Additional file 2: Table S2. Primers used for ChIP-quantitative real-time PCR. [file 12935_2020_1513_MOESM2_ESM.docx]

| **Table S2. Primers used for ChIP-quantitative PCR** | | | |
| --- | --- | --- | --- |
| **Gene** | **primer** | **GeneHancer (GH) Identifier** | **TSS distance (kb)** |
| MMP7-F1 | AGTAGAGCCATGGAAAGCGG | [GH11I102445](http://genecards.weizmann.ac.il/geneloc-bin/display_map.pl?chr_nr=11&range_type=gh_id&gh_id=GH11I102445#GH11I102445) | +79.2 |
| MMP7-R1 | AGCATTGGCTCCAAGGGTTT |  |  |
|  |  |  |  |
| MMP7-F2 | GGGTCATGGAAATCCCCGAG | [GH11I102311](http://genecards.weizmann.ac.il/geneloc-bin/display_map.pl?chr_nr=11&range_type=gh_id&gh_id=GH11I102311#GH11I102311) | +213.1 |
| MMP7-R2 | TAGGGGAACTCCAGCGGTAA |  |  |
|  |  |  |  |
| HMGA2-F1 | CGCTGTAATTTCCAAGCCGC | [GH12I065821](http://genecards.weizmann.ac.il/geneloc-bin/display_map.pl?chr_nr=12&range_type=gh_id&gh_id=GH12I065821#GH12I065821) | +2.3 |
| HMGA2-R2 | GCTCTCGGACAACTTTGGGA |  |  |
|  |  |  |  |
| HMGA2-F2 | AGAAACTCAGCCGCTCTGAC | [GH12I065888](http://genecards.weizmann.ac.il/geneloc-bin/display_map.pl?chr_nr=12&range_type=gh_id&gh_id=GH12I065888#GH12I065888) | +70.8 |
| HMGA2-R2 | TAGCACTGTCTGGCCCCTAA |  |  |
|  |  |  |  |
| CAP1-F1 | AAGGAGTACGGAAGCGAAGC | GH01I040036 | -0.6 |
| CAP1-R1 | ACCCACTCTGCTGAAATCCG |  |  |
|  |  |  |  |
| CAP1-F2 | CAGATGAATGCCGACGCTTG | [GH01I040379](http://genecards.weizmann.ac.il/geneloc-bin/display_map.pl?chr_nr=01&range_type=gh_id&gh_id=GH01I040379#GH01I040379) | +348.5 |
| CAP1-F2 | GTGTAGGCGTGTAGGGACTG |  |  |
