# Supplementary material for: A novel role of Krüppel-like factor 8 as an apoptosis repressor in hepatocellular carcinoma
Source: Cancer Cell Int. 2020 Aug 28;20:422. doi: 10.1186/s12935-020-01513-3 (PMC7456055; doi:10.1186/s12935-020-01513-3)
Supplement: Supplementary file 3 — Additional file 3: Figure S1. Expressions of KLF8 protein in the indicated cell lines were examined by Western blot assay. (A) Western blot analysis of KLF8 in KLF8KO-LM3 or LM3 cells using an anti-KLF8 antibody. (B) Western blot analysis of KLF8 in Huh-7 cells transfecting Flag-tagged KLF8 overexpressing lentivirus or control lentivirus using an anti-flag antibody. GAPDH was used as a loading control. Figure S2. Proliferation of the indicated cell lines was evaluated by determining the cell viability with Cell Counting Kit 8 Assay according to manufacturer’s instructions. The KLF8OE stably transfected Huh-7 cell line and its negative control cell line (CtrlOE) were established using lenti-virus system. The KLF8KO LM3 cell line was established using CRISPR/Cas9 technique. [file 12935_2020_1513_MOESM3_ESM.docx]

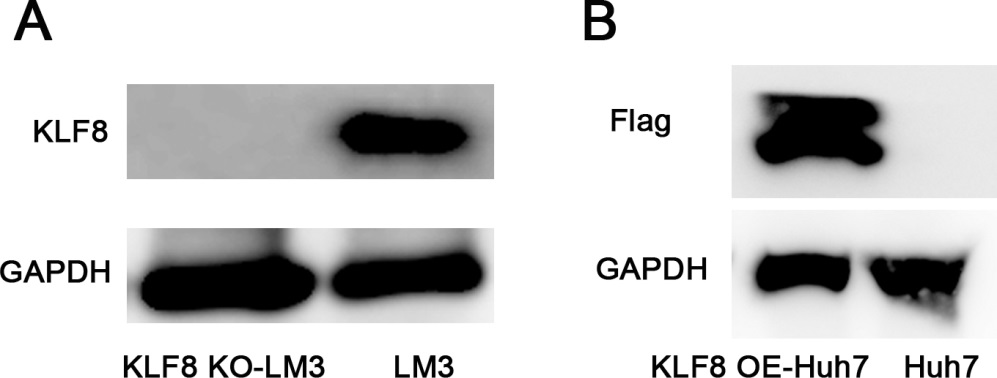


**Figure S1**. Expressions of KLF8 protein in the indicated cell lines were examined by Western blot assay. (A) Western blot analysis of KLF8 in KLF8^KO^-LM3 or LM3 cells using an anti-KLF8 antibody. (B) Western blot analysis of KLF8 in Huh-7 cells transfecting Flag-tagged KLF8 overexpressing lentivirus or control lentivirus using an anti-flag antibody. GAPDH was used as a loading control.


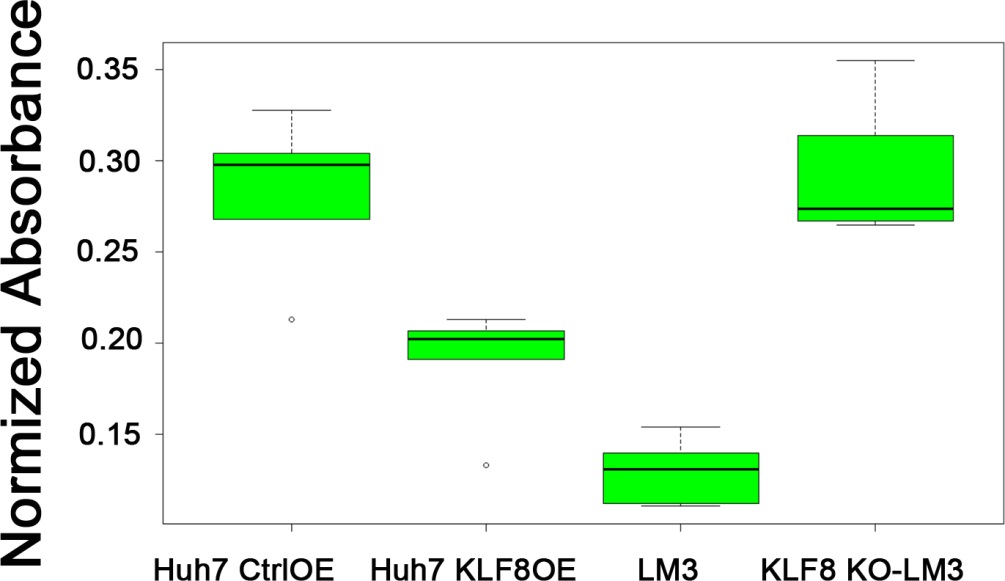


**Figure S2.** Proliferation of the indicated cell lines was evaluated by determining the cell viability with Cell Counting Kit 8 Assay according to manufacturer’s instructions. The KLF8^OE^ stably transfected Huh-7 cell line and its negative control cell line (Ctrl^OE^) were established using lenti-virus system. The KLF8KO LM3 cell line was established using CRISPR/Cas9 technique.
